# Forecasting influenza hospital admissions within English sub-regions using hierarchical generalised additive models

**DOI:** 10.1038/s43856-023-00424-4

**Published:** 2023-12-20

**Authors:** Jonathon Mellor, Rachel Christie, Christopher E. Overton, Robert S. Paton, Rhianna Leslie, Maria Tang, Sarah Deeny, Thomas Ward

**Affiliations:** 1 UK Health Security Agency, Data Analytics and Surveillance, 10 South Colonnade, London, United Kingdom; 2 University of Liverpool, Department of Mathematical Sciences, Liverpool, United Kingdom

**Keywords:** Influenza virus, Epidemiology

## Abstract

**Background:**

Seasonal influenza places a substantial burden annually on healthcare services. Policies during the COVID-19 pandemic limited the transmission of seasonal influenza, making the timing and magnitude of a potential resurgence difficult to ascertain and its impact important to forecast.

**Methods:**

We have developed a hierarchical generalised additive model (GAM) for the short-term forecasting of hospital admissions with a positive test for the influenza virus sub-regionally across England. The model incorporates a multi-level structure of spatio-temporal splines, weekly cycles in admissions, and spatial correlation. Using multiple performance metrics including interval score, coverage, bias, and median absolute error, the predictive performance is evaluated for the 2022-2023 seasonal wave. Performance is measured against autoregressive integrated moving average (ARIMA) and Prophet time series models.

**Results:**

Across the epidemic phases the hierarchical GAM shows improved performance, at all geographic scales relative to the ARIMA and Prophet models. Temporally, the hierarchical GAM has overall an improved performance at 7 and 14 day time horizons. The performance of the GAM is most sensitive to the flexibility of the smoothing function that measures the national epidemic trend.

**Conclusions:**

This study introduces an approach to short-term forecasting of hospital admissions for the influenza virus using hierarchical, spatial, and temporal components. The methodology was designed for the real time forecasting of epidemics. This modelling framework was used across the 2022-2023 winter for healthcare operational planning by the UK Health Security Agency and the National Health Service in England.

## Plain language summary

Seasonal influenza causes a burden for hospitals and therefore it is useful to be able to accurately predict how many patients might be admitted with the disease. We attempted to predict influenza admissions up to 14 days in the future by creating a computational model that incorporates how the disease is reported and how it spreads. We evaluated our optimised model on data acquired during the winter of 2022-2023 data in England and compared it with previously developed models. Our model was better at modelling how influenza spreads and predicting future hospital admissions than the models we compared it to. Improving how influenza admissions are forecast can enable hospitals to prepare better for increased admissions, enabling improved treatment and reduced death for all patients in hospital over winter.

## Abstract (web summary)

Mellor et al. introduce a new method for forecasting hospital admissions with seasonal influenza in a resurgent season that can be used to inform policy makers. The developed generalized additive model shows improved performance over other time series approaches when scored using probabilistic methods.

## Introduction

Seasonal influenza places a substantial burden in the winter months on the primary and secondary healthcare systems in England^1–6^. The recent winters of 2020-2021 and 2021-2022 however, were notable exceptions, with little influenza in circulation, which was likely due to the impact of COVID-19 targeted non-pharmaceutical interventions. Influenza admissions reduce the capacity of the healthcare service to provide emergency care and elective treatment^7^. It is therefore essential that healthcare operational planning are cognisant of the expected demand on the system.

In the United Kingdom, influenza has historically been attributed to around 28,000 hospitalisations annually, with substantial inter-seasonal variation^8^. The disease disproportionately hospitalises young children, the elderly, and adults with high-risk comorbidities^9^. Between the 1996–2009 seasons, weekly hospitalisation rates in the UK were highest in adults over 75 (252 per 100,000), adults aged 65–74 (101 per 100,000) and children under 5 (93 per 100,000)^8^. Being able to forecast hospital admissions with influenza accurately allows clinical administrators to deploy mitigations faster that can curtail system pressures. In the winter 2022–2023, the uncertainty around the likely trajectory of the influenza season and impact of COVID-19 waves on system resources increased the need for fast and flexible models that could be used when population level epidemiological parameters (such as immunity, inter-viral competition, and social mixing behaviour) were less clear. Therefore, forecasting models of influenza hospital admissions, that are less dependent upon predetermined seasonal patterns and estimated epidemiological parameters, would allow public health officials and health system leaders to make informed decisions around policy and response during an irregular season. This includes the deployment of staff, mutual aid between locations, changes to triage or discharge practice, or other methods to increase bed capacity.

Influenza forecasting is a historically well-studied field, with a wide range of methodologies applied^10,11^. Previous work includes a mix of statistical and mechanistic approaches, with the aim of understanding the timing, location, and magnitude of epidemic waves. These include when peaks in infection and hospitalisations occur^12,13^, how large the peaks will be^12^, and incorporating leading indicators^14^. Furthermore, there is a spatial component to the spread of influenza with strong phase correlation based on proximity and connectiveness^15^. This spatial relationship, and the hierarchical nature of spread has been harnessed, such as via mobility data, with either machine learning methods^16,17^ or Bayesian multi-scale approaches^18^. The start of an outbreak is also of interest and has been forecast at fine spatial scales^19^. In the US, many of these policy-relevant metrics are predicted, for example in the Centre for Disease Control (CDC) FluSight forecasting challenge, where a range of models are ensembled^20^. In winter 2022-2023, several factors (uncertainty around population dynamics, the early season, and lack of comparable seasons to train models) meant that a less mechanistic and historically based model is of increased operational utility.

In this paper we present a highly generalisable method for short-term admissions forecasts, which could be applicable to a range of infectious diseases and in a range of settings. It was deployed by UK Health Security Agency in winter 2022–2023. The method could be used nationally, and in local healthcare regions using data within the organisations and using publicly available reference information, without the need for high performance computational resource. The model is a hierarchical generalised additive structure with a spatial component to extrapolate forward 2 weeks the national, regional, and sub-regional trends within England. We score this model’s performance by a range of performance metrics and contrast it with commonly used time series forecasting approaches, highlighting its utility in operational public health response. In this manuscript we show that our model structure out performs other time series forecasting approaches across geographies and forecast horizons.

## Methodology

### Data

An NHS Trust is an organisational unit within the National Health Service (NHS) serving a geographical area or a specialised function; there are 137 acute (containing emergency departments) Trusts in England. Each Trust is within a Sustainability and Transformation Partnership (STP) grouping, formed for NHS Trusts to develop plans to transform healthcare delivery in their shared area^21^, of which there are 44 in England. Each STP has an associated geographic boundary, each within one of the seven NHS England commissioning regions—this structure is outlined in Supplementary Fig. 1. As all data used in this study was aggregated and anonymised, ethical approval was not required. The UK Health Security Agency provided ethics oversight for the study.

### NHSE SitRep and admitted patient care

NHS England (NHSE) influenza data is provided by individual NHS acute Trusts in England, who deliver a daily situation report (SitRep) covering the previous 24 hours on urgent and emergency care activity (UEC) by 11am each day^22^. The reporting process for many Trusts is automated and completed via web form^23^. The data records both hospital bed occupancy and new patients in the past 24 hours with a laboratory-confirmed positive influenza test^24^. We assume positive test patients in the past 24 hours are analogous to admissions throughout this paper, though they likely include hospital acquired infections. Linking information provided in the data includes the Trust, STP^21^, and NHS commissioning region. The data available for this study covers 01 Dec 2021 to the week ending 29 Jan 2023.

The SitRep is used to forecast influenza admissions because it is timely, with limited missing data, and high spatial coverage of England. The SitRep is provided weekly allowing for analysis in real time as the epidemic progresses and is used by the NHS for operational planning. As of 05 Jan 2023, 137 NHS Trusts were reporting data to the SitRep, of which 123 reported data on >99% of days. This provides high coverage of the English population and is representative in all regions. Equally, this allows modelling at NHS Trust or STP level, increasing the operational utility of the approach with granular insight. Furthermore, patients must have laboratory-confirmed positive influenza tests to be included in the SitRep, which increases the reliability of reporting across NHS Trusts, and avoids convoluting counts with other influenza-like-illnesses. Though a high coverage and well specified data source, there are several Trusts which misreported admissions counts due to the rapid turnaround of the reporting and because this is the first reemergent influenza season since the collection started. This can be handled by data cleaning during the season, though it can be challenging to discern between true and false reporting and was partially accounted for in modelling. Trusts that started reporting part way through the season or had clear misreporting were excluded from the analysis.

To support the generalisation of our newly developed model methodology we also produce forecasts using a secondary data set—the Admitted Patient Care (APC) database collected by NHS England^25^. The APC data contains completed individual patient records, including date of admission and ICD-10 diagnosis codes^26^^,^ although there is a multiple month reporting lag. We define influenza admissions as patients with either a primary or secondary diagnosis code in the ICD-10 categories: J09, J10, J11. While local coding practices may vary, admitted patients with influenza diagnosis codes are likely to have been tested for influenza. This gives us an alternative view of the Winter 2022-2023 influenza season in England to the SitRep data which we can validate our model against.

### STP boundaries

When modelling trends in spatial data we expect nearby locations to be closely related. In this case, we expect neighbouring STPs to be correlated. We created an undirected network based on STP boundary adjacency, such that contiguous neighbours are connected and regions that are not adjacent are not connected. This was done such that a Markov Random Field (MRF)^27^ spatial smoothing approach could be included within the modelling. The network itself is unweighted and undirected, the network provides the structure upon which the MRF fits the model coefficients itself.

STP boundary files were obtained from the ONS Open Geography Portal^28^. These contain digital vector boundaries for STPs in England as of April 2021. The STP structure of England, NHS commissioning regions, individual Trust locations and catchment population sizes are shown in Supplementary Fig. 1A, B. The network structure constructed for the MRF component, created via polygon adjacency, is shown in Supplementary Fig. 2. This approach using only neighbours, rather than population mobility connection (which would link London to more areas as a highly connected area) reduces the complexity of the space over which the smoothed intercept is estimated. This is a parsimonious approach using only the data available from the spatial locations available—more complex network structures may better estimate true mobility patterns; however, they rely on more information and stronger assumptions. As the MRF is used to estimate a non-time varying fixed effect in the model we do not anticipate the network structure itself having a substantial impact of forecast performance relative to other model components.

### Respiratory local authority to trust mapping

Larger STPs service correspondingly large population catchments, and therefore should have higher daily counts of influenza patients as the epidemic progresses. We normalised admission counts by the population catchment size for an STP, giving a per capita rate. As the hospital an individual visits depends on location, choice^29^ and age group^30^ we created a probabilistic mapping between lower tier local authority (LTLA) administrative regions and NHS Trusts, linking resident populations and hospitals. This mapping used counts of patient records from Secondary Use Services—All Patient Care^25^, linking a discharge location (LTLA) to the service provider (NHS Trust), in a similar manner to the *covid19.nhs.data* R package^31^. The counts include all patients admitted from 01 Jan 2021 to 11 Dec 2022 with a respiratory-related primary or secondary diagnosis code, excluding COVID-19 patients— using COVID-19 test linkage with the Second Generation Surveillance Service (SGSS)^32^. ONS 2019 local authority population estimates are used to produce a weighted sum of populations across Trusts of their feeder LTLAs, giving an effective population catchment size. Since the data were modelled at STP spatial level for these forecasts, admissions and populations were aggregated from reporting Trusts within an STP to calculate the rate.

### Model

#### Generalised additive model (GAM) structure

To forecast hospital admissions with influenza, we first assumed a semi-mechanistic model. We assumed in an exponentially growing epidemic we have the per-capita rate, with population $p$, of hospitalisations at time $t$, $H(t)/p$,

$$\frac{H\left(t\right)}{p}=\frac{H\left(0\right)}{p}{e}^{{rt}}.$$

Instead of assuming a constant growth rate $r$, we can consider the growth as a smooth function of time $s(t)$, i.e.

$$\frac{H\left(t\right)}{p}=\frac{H\left(0\right)}{p}{e}^{s\left(t\right)}.$$

Such a model can be used to generate short-term forecasts by assuming that the growth rate remains constant outside of the data window, i.e., $s\left(t\right)=r* t$ when $t\ge {t}_{\max }$, where ${t}_{\max }$ is the final date for which we have data. To fit such a model, we need to estimate the smooth function $s\left(t\right)$ for $t\in [0,{t}_{\max }]$. Taking the logarithm of both sides, this becomes

$$\frac{H\left(t\right)}{p}=\frac{H\left(0\right)}{p}{e}^{s\left(t\right)}\Rightarrow \log \left(\frac{H\left(t\right)}{p}\right)=\log \left(\frac{H\left(0\right)}{p}\right)+s\left(t\right).$$

$$\Rightarrow \log \left(H(t)\right)=\log \left(\frac{H\left(0\right)}{p}\right)+s\left(t\right)+\log (p).$$

The number of hospital admissions on each day, $H\left(t\right)$, is an overdispersed integer valued count, which can be considered as a random sample from a negative binomial distribution. Therefore, the smoother $s(t)$ can be estimated as $y(t)$ by using a GAM with negative binomial error structure, with an intercept term ${\beta }_{0}$, smoothing function of time $f\left(t\right)$, and offset for the population size $p$, i.e.

$$\log \left(y(t)\right)={\beta }_{0}+f\left(t\right)+\log (p).$$

#### Incorporating STP level structures and population size

We aggregated the data to STP level, so all sub-regions have at least one reporting hospital Trust, which allows us to create a connected network of adjacent sub-regions. We assumed a seasonal influenza outbreak will have a national epidemic curve component, as well as trends sub-nationally (STP level), but these sub-national trends are not fully independent of one another in shape. A hierarchical GAM was used to account for the group-level structure. Following the GS form from Pedersen et al.^33^, we add a global smoother (national level) and group-level smoothers (STP level) that have the same wiggliness—a “Global smoother with individual effects that have a Shared penalty”^33^. To further account for between-group correlation of adjacent STPs, we estimated an intercept using spatial smoothing, correlating adjacent STPs using a MRF approach, and a network based on adjacency shown in Supplementary Fig. 2.

Each STP has different population catchment sizes, so we included an offset, ${p}_{i}$, to calculate an admissions rate per capita for a given STP $i$; we transform the forecasts back to counts for ease of comparison in reporting. We further included a regional random effect ${\delta }_{{regio}{n}_{i}}$. And as hospital admissions and test reporting are impacted by the day of week (DOW), we include a random effect ${\delta }_{{{dow}}_{t},{st}{p}_{i},{regio}{n}_{i}}$ of DOW by STP nested within region. As reporting quality varied between STP and region the DOW random effect is fitted for each STP, and for those STPs with limited information on effect size, this term converges to the regional effect. The STP intercept was approximated by the Markov Random Field ${f}_{{mrf}}\left(i\right)$, the STP temporal trends by the individual effect shared penalty ${f}_{{st}{p}_{i}}\left(t\right)$ and a national trend by ${f}_{{nat}}\left(t\right)$. This gives the model formulation as

$$\log \left({y}_{i}(t)\right)= 	\, {\beta }_{0}+{f}_{{nat}}\left(t\right)+{f}_{{st}{p}_{i}}\left(t\right)+{f}_{{mrf}}\left(i\right) \\ 	+{\delta }_{{{dow}}_{t},{st}{p}_{i},{regio}{n}_{i}}+{\delta }_{{regio}{n}_{i}}+\log ({p}_{i}).$$

Forecasts at multiple geographies are policy relevant: individual NHS STPs, regions, and nationally. To obtain these, we aggregated the individual time series forecasts using a “bottom up” approach, where disaggregate predictions are aggregated to higher levels, such as STP to region and to nation. This is often more accurate when disaggregate data is available than “top down” methods, where data is first aggregated to a high level, forecasts generated, then disaggregated proportionally to the lower levels^34,35^. Where STP level count data is weak, the model is still accounting for national trends, which partially mitigates low data quality in individual STPs.

This hierarchical structure, with shared parameters between national and STP level splines defining the model structure, reduces the total flexibility in the model—as fewer parameters are penalised. In addition, as the temporal splines are defined by the number of basis functions, the number of basis functions sets the level of flexibility in the model, and its propensity to overfitting—for which the model sensitivity can be explored.

#### Implementing the model

We implemented the GAM using the R package *mgcv*^36^. In *mgcv*, the negative binomial distribution is parameterised in the terms of the mean, $\mu$, and variance, ${\sigma }^{2}$, such that $\mu =E\left[y\right]$ and ${\sigma }^{2}=E\left[y\right]+\frac{E{\left[y\right]}^{2}}{\theta }$. In this formulation, the *θ *parameter is fitted by the package rather than specified by the user. We selected thin plate splines as the basis functions, with the optimum number of basis functions chosen by scoring different combinations. Sensitivity analysis was conducted to understand the optimal model structure formed by the different additive components and hierarchical relationships during model development. In *mgcv*, the parameter $k$ sets the dimensionality of the spline and so the number of basis functions is given by $k-1$. We define the number of days per basis function as ${t}_{d}$ and the number of days the model is fit with as ${t}_{{length}}$. We round down the ratio ${t}_{{length}}/{t}_{d}$ to the closest integer to calculate $k-1,$

$$k-1=\left\lfloor \frac{{t}_{{length}}}{{t}_{d}}\right\rfloor .$$

The relationship between ${t}_{d}$ and the number of basis functions is shown in Supplementary Fig. 3. In this analysis ${t}_{{length}}$ is chosen as 63 days (9 weeks) and *t*_*d*_ ∈ 1,14 for both ${f}_{{nat}}(t)$ and ${f}_{{st}{p}_{i}}(t)$. The optimal model is found to be ${t}_{d}=5$—all results are shown for this value for national and STP splines.

#### Baseline ARIMA model

To contextualise the performance of our newly developed hierarchical GAM model we compared it with a commonly used approach. As a baseline, we chose an autoregressive integrated moving average (ARIMA) model^37^, a widely employed technique in time series forecasting in epidemiology^10,38,39^. The structure of an ARIMA is explained in further detail by Nau^40^, though we provide a summary of relevant parameters. A seasonal component is also incorporated, SARMIA, to capture periodic trends (e.g., weekly cycles in admission numbers). $(S){ARIMA}\left(p,d,{q}\right){\left[P,D,Q\right]}_{s}$ describes the model, where $p$ is the number of autoregressive terms, past values of the time series; $d$ is the number of differences of the time series to make the series stationary; and $q$ is the number of lagged forecast errors included. $P,D,Q$ and $s$ stand respectively for seasonal autoregression, seasonal integration, seasonal moving average, and season period length. We transformed the influenza admission counts using $\log (x+1)$ to account for the non-negative, exponential trends of the epidemic. We apply the ARIMA models implementing the Hyndman–Khandakar algorithm within the R package *forecasts*^41^ via *fable*^42^ which determine the characteristic model parameters by minimising the AIC after differencing the data. As with the GAM, we use 9 weeks of historic data to predict a 2-week trend. Regional, national and STP level trends are fit separately rather than aggregating the granular STP forecasts in a bottom-up approach. This differs from the GAM structure as we found that the non-hierarchical structure performed better for the ARIMA model; the scores for the hierarchical and non-hierarchical model can be found in the Supplementary Table 1.

#### Baseline prophet model

To further contextualise the performance of our GAM model we compared its performance to another alternative method commonly used in industry, Prophet, developed by Facebook^43^. Prophet is a time-series modelling R package^44^, which creates additive models of the form

$${y}_{i}\left(t\right)=g\left(t\right)+s\left(t\right)+h\left(t\right)+{\epsilon }_{t},$$

with $g\left(t\right)$ defining the trend, $s\left(t\right)$ the periodic changes (e.g. seasonality), $h\left(t\right)$ an effect for irregularly placed holidays and ${\epsilon }_{t}$ the error term^45^. While the model is intended for use with business data, it has been applied to epidemic forecasting during the COVID-19 pandemic^46,47^, and to other diseases, such as hand, foot and mouth^48^. As with the GAM and ARIMA we fit the model to the past 9 weeks of data to predict the coming fortnight. We fit separately at national, regional and STP geographic levels. To account for the non-negative exponential trend, we again apply a $\log (x+1)$ transformation to the influenza admission counts. Prophet allows for daily, weekly, and yearly seasonality—we allowed for weekly seasonality due to the daily granularity of data and sub-year length of data fit to.

#### Model evaluation

The quality of each model was assessed by quantifying the calibration, sharpness, over and underprediction, bias, and median absolute error (MAE) of the predictions. Calibration is defined as the statistical consistency of the forecasts and true observations, for which we chose the 50% and 90% interval coverages generated from 1000 prediction samples. Bias, as well as overprediction and underprediction, refers to whether the predictions are higher or lower than the true response values. The (Weighted) interval score was chosen as it combines over and underprediction, along with sharpness, a measure of the model’s concentration of the predictive distributions-how wide its intervals are^49,50^. The MAE provides a more intuitive number to explain to end users of the forecast as it is in the natural units of the data presented with the forecast. Scoring metrics were implemented with the *scoringutils* R package^50^. We forecasted up to 14 days and evaluated weekly predictions to replicate a real-world application. The performance was examined from the start to end of the winter 2022-2023 seasonal epidemic wave. The predictive performance at STP, regional and national levels are examined for the $t\in [{t}_{\max }+1,{t}_{\max }+14]$ forecast horizon, as policy decisions are often made at higher geographies. Where appropriate, we scored the predictions at ${t}_{\max }+7$ and ${t}_{\max }+14$ to highlight differences in the length of the forecast horizon in different models. The MAE and bias are reported over time for each prediction to examine the trend across the influenza season.

### Reporting summary

Further information on research design is available in the Nature Portfolio Reporting Summary linked to this article.

## Results

### Influenza season 2022–2023

**Fig. 1:**
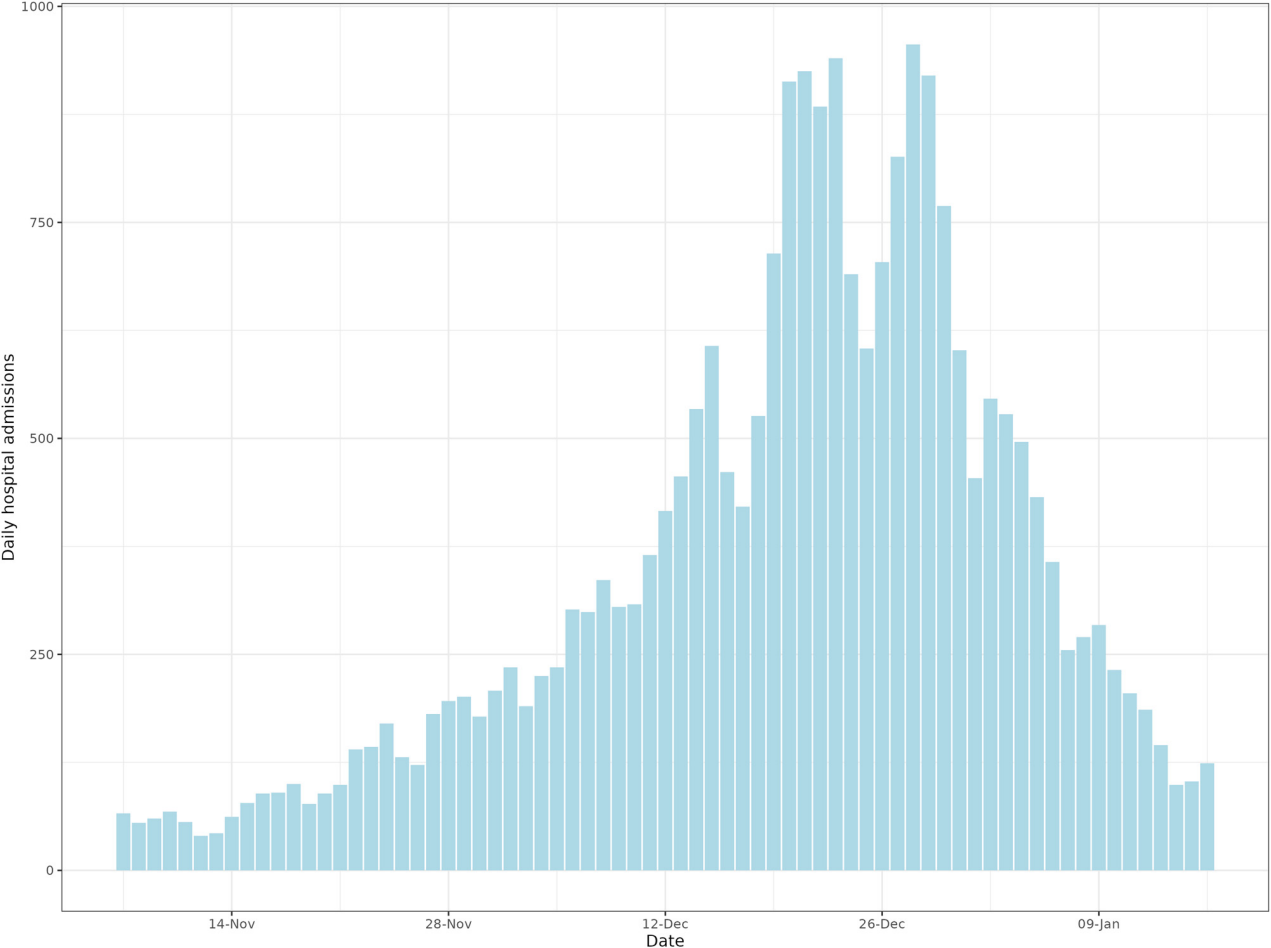
National influenza hospital admissions 2022-2023 season in England. Data from the National Health Service England (NHSE) Urgent and Emergency Care Situational Report, between 18 Sept 2022 and 29 Jan 2023.

The 2022–2023 influenza season saw hospital admissions in England increase rapidly at the start of November and reached a peak by the end of December, after which the number of cases admitted to hospital decreased rapidly, shown in Fig. 1. The data from the NHSE UEC SitRep suggested that the peak would have occurred between the 21 Dec 2022 and 29 Dec 2022 at ~1000 new patients per day, a period of atypical social mixing patterns. However, there were likely data reporting issues and changes to triage behaviour caused by bank holidays, which could have contributed to the decrease in cases reported within that period. From an operational perspective the timing of this peak, just before most surveillance systems were at reduced capacity makes this epidemic wave a particular challenge to forecast, as data is less timely and fewer complimentary data sources are available to validate trends. Additionally, the day of week effect observed in the data was compounded by the timing of bank holidays, for example, Christmas day fell on a Sunday, the day of the week with lowest expected admissions—reducing the clarity of the epidemic structure. The individual STP admission trends are shown in Supplementary Figs. 4–11, highlighting the variation in size of wave and consistency of reporting.

### Weekly forecasts

#### SitRep data

**Fig. 2:**
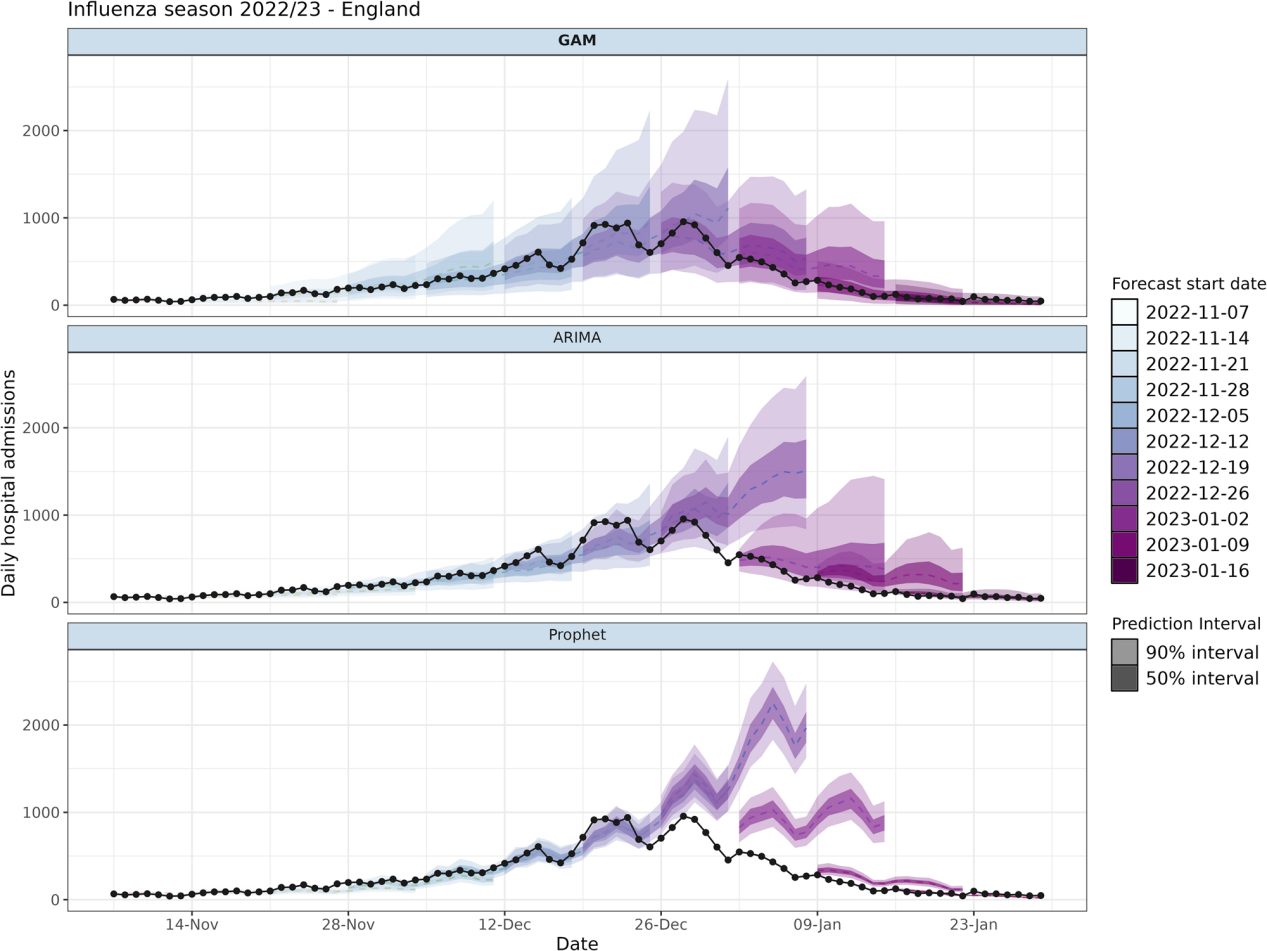
Weekly admissions forecasts of the influenza season in England 2022-2023. Weekly forecasts of a 14-day horizon for the GAM (bolded text), ARIMA and Prophet models. Black dots and black lines represent the true admissions on a day. The filled colours correspond to the week of forecast production.

The national forecasts of the GAM, ARIMA and Prophet models are shown in Fig. 2, for the 2022–2023 season in weekly intervals, illustrating the performance at different phases of the epidemic wave. The national forecast shows that the GAM was quicker to identify the turning point at the peak of the epidemic wave, indicated by the forecasts starting 19 Dec 2022 to 02 Jan 2023. The ARIMA model continued the previously observed trend, as expected from an autoregressive model, causing it to overshoot at the peak, and overpredict on the subsequent decay in admissions. However, the ARIMA performance appeared strong in the initial growth phase when the growth rate is more consistent. The Prophet model too overshoots the epidemic peak and was slower to correct on the decline phase compared to the ARIMA. The prediction intervals for the GAM were generally wider than the ARIMA and substantially more than the Prophet models, which captured more overdispersion and variation in weekly reporting patterns. The central estimate of the GAM model near the epidemic peak had a better fit to the data, with reduced underpredictions in the growth phase, and smaller overprediction in the decline, despite this also being an autoregressive model. Individual STP level forecasts are given in Supplementary Figs. 12–18.

**Fig. 3:**
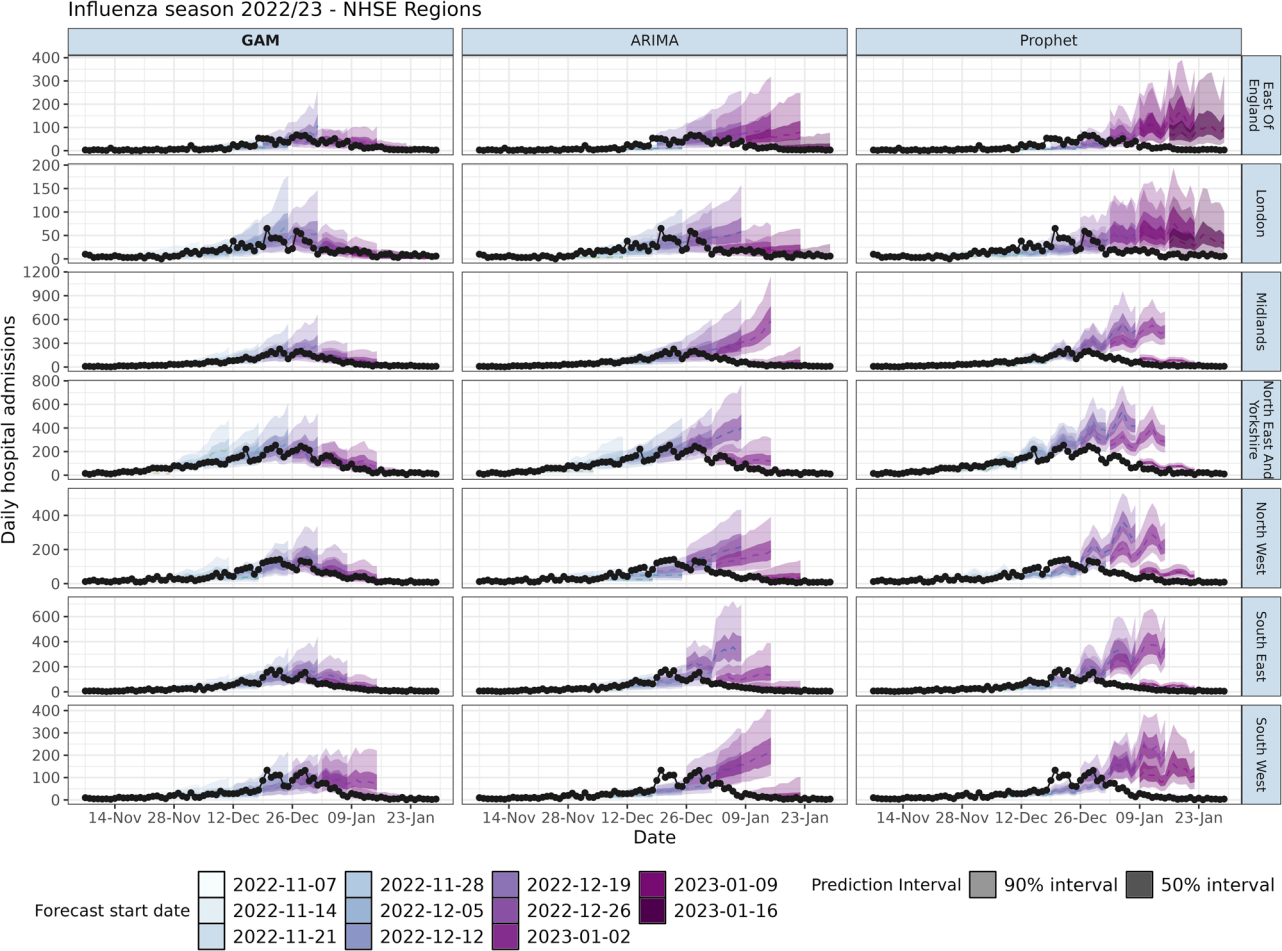
Weekly admissions forecasts of the influenza season in England 2022-2023 by commissioning region. Weekly forecasts of a 14-day horizon for the GAM (bolded text), ARIMA and Prophet models. Black dots and black lines represent the true admissions on a day. The filled colours correspond to the week of forecast production. Note that the y-axis differs for each region.

The superiority of the GAM extends to the regional modelling of England, as demonstrated in Fig. 3. In multiple regions the ARIMA struggles to adapt to the change in direction at the epidemic peak, such as in the South West, where consecutive forecast intervals are non-overlapping. However, the GAM model also struggled to fit the epidemic decay phase in the South West as well as other regions in the week commencing 02 Jan 2023. For some regions, such as the South East, the ARIMA model has substantially wider prediction intervals. In many weeks Prophet did not appear to fit well to the most recent (and impactful) data, such as on 09 Jan 2023, there is a large gap between the forecast ${{y}_{i}}(t_{\max }+1)$ and the data ${H}_{i}({t}_{\max }+1)$ in the South West. From 09 Jan 2023 onwards, the GAM detected a decline and reduced the width of the prediction intervals, however the ARIMA struggled to do this at a regional level, instead predicting 90% intervals that included growth, shown clearly in the Midlands. The Prophet model appeared to forecast the shape of the epidemic, though weeks after it occurs, such as in the later weeks in London. The models were fit to data of varying quality, which was illustrated by the low counts observed in London and the East of England, which were indicative of low ascertainment rates or poor reporting. The hierarchical component of the GAM allowed for the national smoother to inform the forecasts, pooling the trend from all STPs which helped to improve performance relative to the independent fits of the ARIMA and Prophet approaches.

#### Admitted patient care data

The ability of our GAM method to capture the epidemic trend is not limited to the rapid turnaround SitRep data used operationally for this work alone. We applied the same model to the time-lagged Admitted Patient Care influenza admissions 2022 wave to validate its ability is not just an artefact of the rapid situational report data alone. The weekly forecasts are shown in Supplementary Figs. 20, 21. The APC data for 2022 has a similar national and regional shape to the SitRep, however, the counts are higher giving a more pronounced shape. Applied to this data, the GAM captures the direction of the epidemic trend during both growth and decline, although as with the SitRep there is still overprediction at the peak, the GAM does quickly adapt.

### Comparative performance

#### Performance over time

**Fig. 4:**
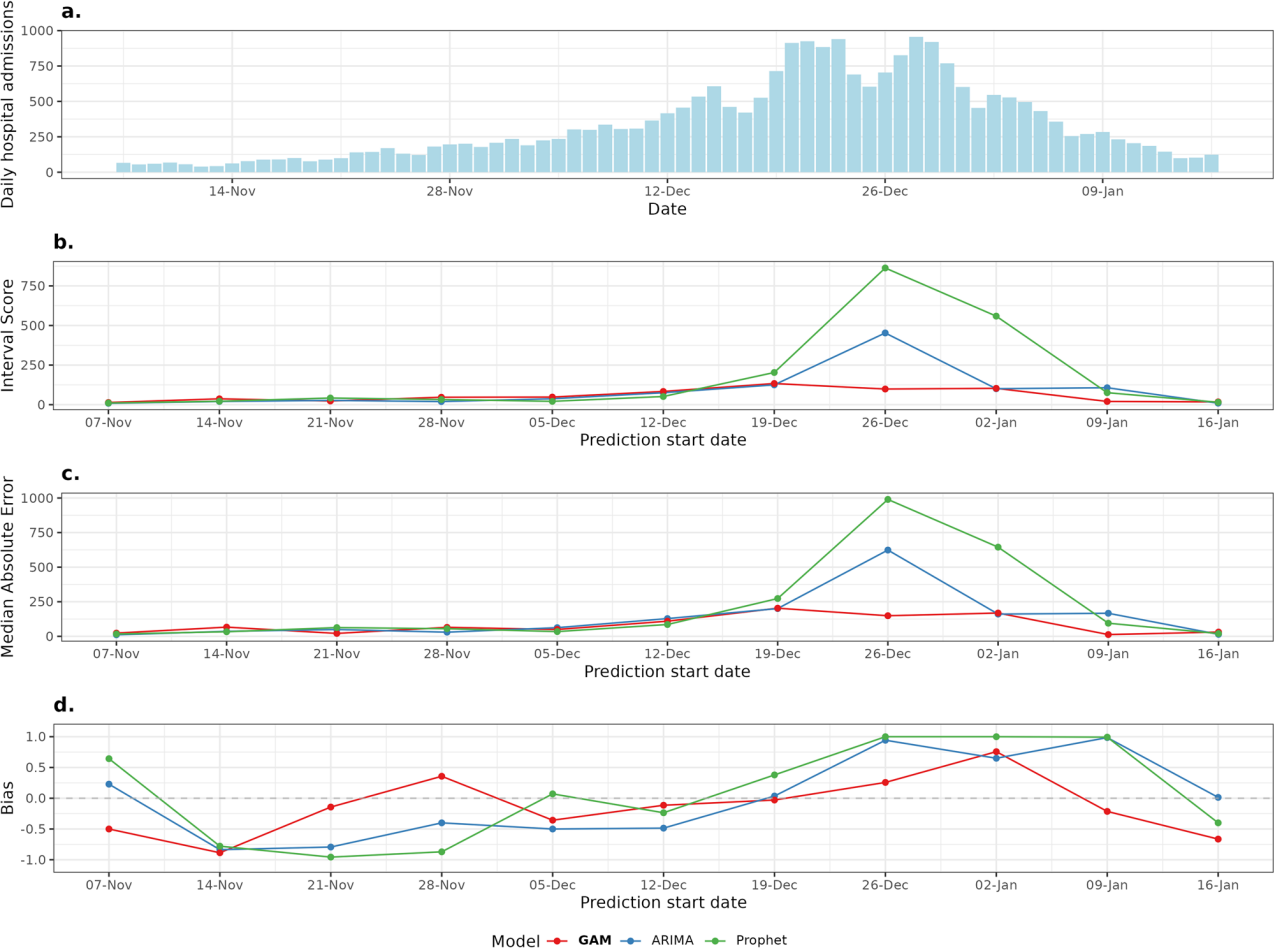
The epidemic wave and corresponding GAM, ARIMA and Prophet model performances for forecasts starting each week. Performance across a range of metrics for the GAM (red line and point) ARIMA (blue line and point) and Prophet (green line and points). **a** The counts (blue columns) of daily hospital admissions within the English 2022-2023 winter season. **b** Interval score for each week of forecasts showing the calibration over time. **c** Median Absolute Error for each week of forecasts showing the error over time. **d** Bias for each week of forecasts showing the under/overprediction over time.

The out-of-sample performance for the interval score, MAE and bias are shown as the epidemic wave progresses through time for each model in Fig. 4. Across most weekly forecasts, the GAM’s bias was closer to zero than the ARIMA and Prophet. The GAM’s bias was further from zero at the very beginning and end of the time series, in early growth and decline phases where counts are lowest. The ARIMA and Prophet models underpredicted during the epidemic growth phase then switched to overprediction at the peak and beyond. This under then over prediction effect was more pronounced in the Prophet model than the simpler ARIMA. The MAE and interval score of each model is broadly consistent during the epidemic growth phase, but the scores diverged sharply at the epidemic peak, as shown in Fig. 2 where the Prophet and ARIMA models overpredicted the peak and then struggled to identify the decay phase in a timely fashion. The interval score in this case was the preferred measure of performance to account for sharpness, how concentrated the intervals are, and under/overprediction.

#### Forecast horizon and geography

The hierarchical GAM introduced in this study consistently outperformed the ARIMA and Prophet models across a wide range of scoring metrics, different geographies, the whole forecast horizon, and for both ${t}_{\max }+7$ and ${t}_{\max }+14$, shown in Table 1. The national and regional forecasts were of greatest interest to public health officials—where the GAM outperformed the Prophet and ARIMA with a lower MAE and better calibration (interval score). Understanding whether the models were likely to over or under predict admissions was also important when disseminating the estimates to decision makers and for both metrics the GAM scored better than Prophet and the ARIMA at coarse geographies. Although, the ARIMA has a better underprediction score at national level for ${t}_{\max }+14$. For all geographies the ${t}_{\max }+14$ GAM outperformed the ${t}_{\max }+7$ Prophet model. The ARIMA performed best for the 50% coverage, whereas the GAM scored best for 90%—as the GAM had generally wider interval coverage particularly for ${t}_{\max }+7$. In some cases the Prophet and the ARIMA had very low coverage which indicated they overconfidently get the direction of the epidemic wrong with coverage below 0.300.

**Table 1:** Interval score, underprediction, overprediction, absolute median error and coverage at 50% and 90% for the ARIMA, GAM and Prophet models at national, regional and STP level.

Evaluation scores for ARIMA, GAM and Prophet models							
Model	Horizon	Interval Score	Underprediction	Overprediction	Median Absolute Error	50% Coverage	90% Coverage
National							
GAM	Overall	**57.0**	**9.20**	**12.7**	**81.8**	0.714	**0.987**
ARIMA	Overall	89.5	12.4	52.5	135	**0.409**	0.805
Prophet	Overall	172	12.9	146	210	0.195	0.468
GAM	7	**42.9**	**3.14**	**9.53**	**60.3**	0.818	1.000
ARIMA	7	71.4	3.43	47.6	107	**0.364**	**0.818**
Prophet	7	138	10.5	117	170	0.091	0.455
GAM	14	**108**	10.4	**49.4**	**170**	0.364	**0.909**
ARIMA	14	176	**8.44**	130	253	**0.455**	0.636
Prophet	14	308	24.8	267	359	0.182	0.182
Regional							
GAM	Overall	**10.1**	**2.38**	**2.69**	**15.4**	**0.613**	**0.944**
ARIMA	Overall	21.0	4.46	11.0	31.2	0.350	0.745
Prophet	Overall	35.0	4.90	24.9	47.5	0.230	0.524
GAM	7	**8.07**	**1.31**	**2.43**	**11.8**	0.675	**0.948**
ARIMA	7	17.6	2.27	10.4	26.5	**0.364**	0.740
Prophet	7	26.6	3.60	18.8	37.3	0.143	0.532
GAM	14	**17.8**	**2.53**	**8.43**	**28.3**	**0.416**	**0.831**
ARIMA	14	37.4	4.73	24.6	53.4	0.221	0.623
Prophet	14	52.6	6.14	40.5	68.1	0.169	0.390
STP							
GAM	Overall	**2.770**	**1.030**	**0.912**	**4.200**	**0.579**	**0.844**
ARIMA	Overall	4.160	1.580	1.380	6.030	0.365	0.679
Prophet	Overall	7.100	1.780	3.960	10.000	0.310	0.564
GAM	7	**2.250**	**0.665**	**0.863**	**3.400**	0.617	**0.864**
ARIMA	7	3.530	1.030	1.400	5.220	**0.385**	0.699
Prophet	7	5.830	1.260	3.370	8.350	0.297	0.576
GAM	14	**4.180**	**0.969**	**2.070**	**6.370**	**0.535**	**0.775**
ARIMA	14	6.210	1.660	2.780	8.760	0.314	0.641
Prophet	14	9.860	1.810	6.460	13.300	0.271	0.491

The Horizon column denotes how each model scores for the prediction at 7 and 14 days and across all predictions in the 2-week forecast, from each week starting 07 Nov 2022 to 16 Jan 2023. The score in bold highlights the best score of the models for each horizon. Bold elements represent the best scoring value for a model within a geography and horizon.

#### GAM spline tuning

**Fig. 5:**
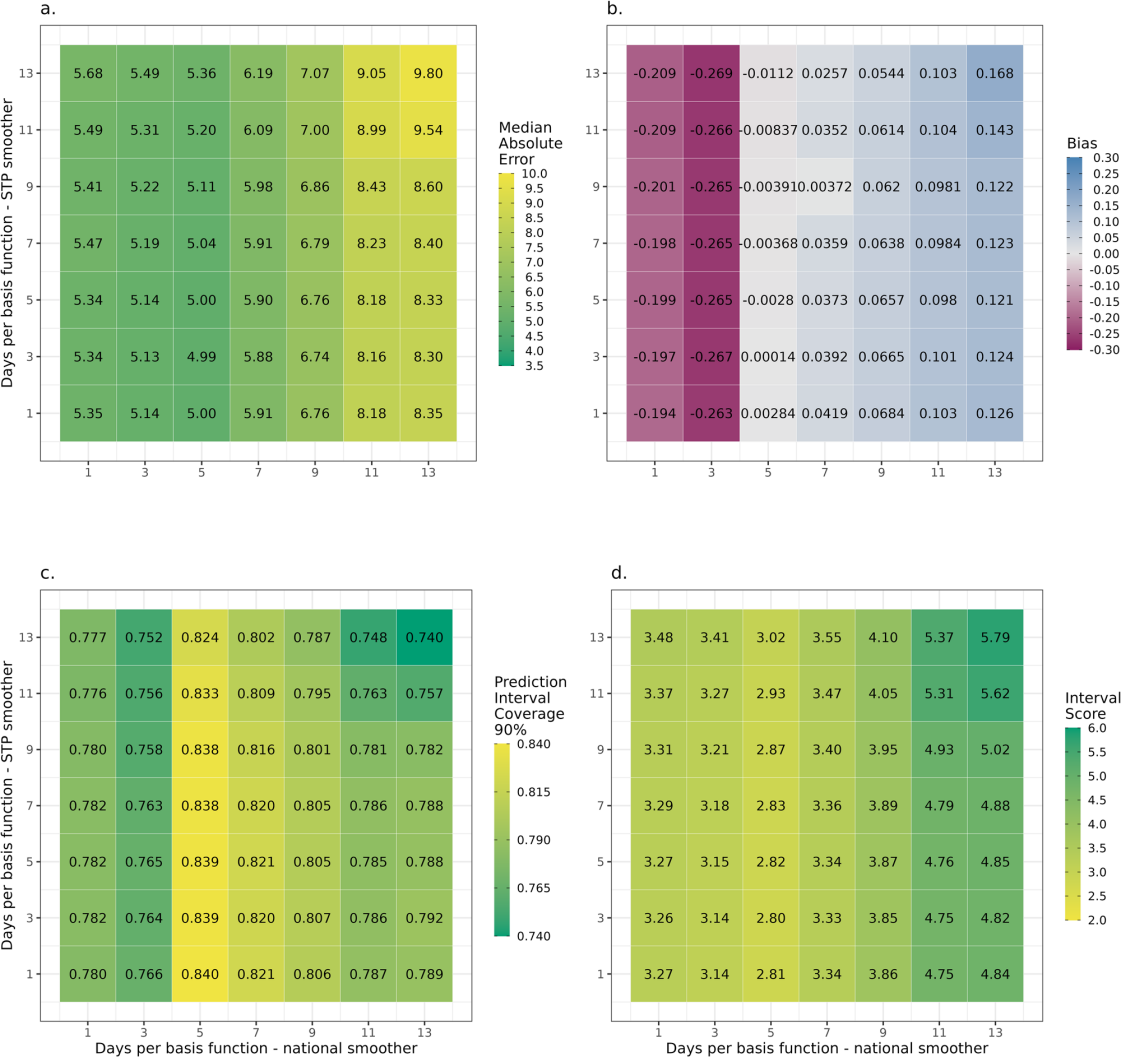
Scoring for a range of GAM spline parameters used to select the best performing combination. **a** The median absolute error (**b**) bias, (**c**) prediction interval coverage and (**d**) interval score metrics for combinations of different numbers of basis functions in the hierarchical GAM. Models were evaluated on held out forecasting performance for 2-week projections, each week from 10 Nov 2022 to 08 Jan 2023.

GAMs use splines to model smooth relationships between response and explanatory variables. The flexibility of splines to fit non-linearities in response variables is determined by the number of basis functions in each smoothing term, with more basis functions allowing for more non-linear relationships to be modelled, but at the increased risk of modelling noise as signal. The amount of smoothing is crucial to the model’s predictive power; therefore, a sensitivity analysis was performed on the model’s two main parameters. The performance of the different GAM parameters is shown in Fig. 5 for multiple scoring metrics and a range of days per basis spline—for the national, ${f}_{{nat}}\left(t\right)$, and STP level ${f}_{{st}{p}_{i}}\left(t\right)$ smoothers. This showed the out-of-sample performance of the forecasts were highly dependent on ${d}_{t}$ for the national smoother (more variation along the x-axis), and to a lesser extent the STP smoother (less variation along the y-axis). The best performance of the national smoother occurred at 5 days per basis function consistently, though the best score for the STP smoother varies across metrics. As the bias metric was aggregated across the forecast horizon and epidemic wave the bias shown represented the average bias, which may be near zero due to both over and under prediction over the study period. The performance for all integer numbers of basis functions *t*_*d*_ ∈ 1,7 are available in Supplementary Fig. 22 to show more granularity nearer the optimal values.

## Discussion

In this study we show that a hierarchical GAM is an effective approach to short-term forecasting of influenza hospitalisations across England, outperforming the commonly used statistical ARIMA and machine learning Prophet approach. The model performed well both at low spatial levels (STP) and higher (region and national) aggregations. The GAM is impacted less by the expected issues of an autoregressive model at an epidemic peak, was less biased over time, and had a lower interval score than Prophet and the ARIMA at both 7 and 14 days into the future. The model was sensitive to the number of basis functions in a smoother, particularly in the national component of the multi-level model, which highlighted the need for careful parameter tuning during a season.

The hierarchical GAM introduced in this study had several strengths, making it a useful addition to the research in forecasting healthcare burdens due to infectious diseases. This modelling approach is easily applicable to other illnesses, does not rely on strong assumptions based on past seasonal patterns or epidemiological parameters and is robust when data quality may be low. Mechanistic models of influenza transmission and hospitalisation rely on detailed parametrisation (e.g., contact rates, infection hospitalisation rate, incubation, and infectious period) and particular initial conditions (e.g., susceptible population, current prevalence), which this approach avoids. Exploring the pairwise combinations of different parameters and scenarios is computationally costly and contentious, as those providing model evidence during the COVID-19 pandemic can attest^51^. By taking a data-driven semi-mechanistic approach, we principally depend on subtle signals at fine spatial scales to detect upcoming changes in rates, and the computational efficiency of our method allows for weekly updates to projections in a highly localised level. The computational requirements of these models are low compared to mechanistic models; they can be run on a standard laptop producing forecasts in under 15 minutes. From receiving the data to sharing the analysis with decision makers, the main time limitations are the quality assurance and dissemination, rather than the time to produce outputs.

By limiting the forecast horizon to 14-days this model avoids overextrapolation while still having high predictive performance and meeting the needs of policy makers. The spatial structure of the model, correlating adjacent STPs, and pooling the temporal trends through the hierarchical structure allows the model to detect the slowing growth rate near the peak of a wave, taking advantage of the spatial variation in influenza peak times^52^. The explicit encoding of a nested random effect for the day-of-week effect allows the capturing of reporting and presentation patterns which the independent spatial structures of the ARIMA and Prophet models struggle to capture. Due to the nature of basis functions, the GAM is impacted most severely by the most recent data fit to, allowing it to capture short-term changes, such as a recent epidemic peak and changes in growth rate.

While the motivation of the structure of this model was to suit the policy needs and NHS SitRep data supplied, this approach is generalisable to data rich surveillance systems. We showed in Supplementary Figs. 20 and 21 that this model approach works well on an alternative data set, the retrospective Admitted Patient Care. Although, further improvements could be made retuning the basis function parameters specifically for this data. The APC data is higher quality (due to the financial incentives and time delay involved) than the SitRep, especially at fine spatial scales, with less stochastic noise associated. Tuning the model’s main parameters to this data would increase its predictive performance by selecting the spline lengths that best characterise the change in growth/decay of the wave, which would match the assumption of constant growth rates better. Evaluation of a probabilistic forecast is crucial to the operational use of a model. We employed robust scoring rules^53^, particularly the interval score, to show the model calibration and out-of-sample performance in an unbiased and consistent way for quantile forecasts^53,54^.

While this study introduced a newly developed and clearly effective technique for forecasting infectious disease hospitalisations—there were challenges associated with the approach. The reliance on fast turnaround granular situational report data sources were crucial to short term forecasting, however these can often have data quality issues requiring ad hoc fixes. Without these corrections a spline-based model may over interpret inaccurate signals, which can cause incorrect predictions due to the responsiveness of the splines. When deploying a modelling approach at the beginning of a novel epidemic season it cannot be known what the optimal parameter values over the whole wave will be, though consistent scoring with each new week of data helped mitigate this issue. What we cannot know until the next influenza season is whether this method performed well because of the pronounced epidemic shape of the influenza wave, which presented a clear growth then decay phase. Forecasting a season with a longer flatter plateau or several phases of growth and decay could be more challenging for this method, but the spatial and hierarchical components should aid model performance. Future work should apply this method to future seasons which will have different shapes to validate these results. This is not possible to do for historic waves as there are no high coverage test-positive daily admissions data sets in England pre-2021. The prediction intervals of this method are wide, which does introduce challenges for policy decisions, though this is also the case for the ARIMA model. An improved method for aggregating uncertainty from low level prediction samples could improve model confidence. However, as is evidenced by the performance of the Prophet model, projections with thin prediction intervals, but highly inaccurate predictions, could falsely give policy makers confidence in incorrect forecasts. Furthermore, the evaluation of this approach could be improved by incorporating the exponential structure into the scoring metrics^55^. Now that there has been a seasonal influenza wave, the time invariant spatial components of this epidemic wave, such as population demographics and geographic features should be explored and incorporated for the next season. In addition, the inclusion of leading indicators such as community testing, syndromic surveillance, or age structures, could improve the predictive performance and confidence of the model. Although, this does increase the complexity of the approach and number of dependencies required for real-time forecasting.

A core assumption of our model is that the estimated growth rate at the end of the observed time series holds for the following 2 weeks. However, naively calculated growth rates using poorly penalised splines, which often display overfitting behaviour for extrapolation tasks, will confound across this forecast horizon resulting in inaccurate predictions. The strength of our method is to disaggregate to a sensible spatial scale, to allow for differences in the timing and magnitude of changes to growth rates at the local scale to be reflected in estimates for other geographies (through the hierarchical terms in the model). Our analysis has shown this has clear performance benefits over other methods. However, ideally the model would anticipate and include changes to the growth rate that occur within the projected 2-week interval. This could be achieved by including leading indicators of influenza admissions which could reflect upcoming changes in growth rate before they were reflected in admissions, as has been shown for models of COVID-19 admission^56^.

In this study we have introduced a newly developed method for short-term forecasting of influenza admission waves, applied in real time during the England 2022–2023 influenza season. The hierarchical structure, spatial components, and spline-based approach to capturing epidemic trends were shown to substantially out-perform commonly used time-series approaches across a range of scoring metrics and forecast horizons. The hierarchical GAM shown is simple to apply relying only on past time series and open-source reference data, without strong assumptions on population immunity levels, vaccinations, or disease strains. The hierarchical and spatial components allow the model to perform well at peak times comparatively to a purely autoregressive models by adding epidemic relevant domain knowledge. Further study into age structures of disease and leading indicators would perhaps improve model confidence, while coming at the cost of simplicity.

### Supplementary information

Supplementary Information

Reporting Summary

## Data Availability

UKHSA operates a robust governance process for applying to access protected data that considers: the benefits and risks of how the data will be used compliance with policy, regulatory and ethical obligations, data minimisation. In addition, how the confidentiality, integrity, and availability will be maintained, retention, archival, and disposal requirements. As well as best practice for protecting data, including the application of ‘privacy by design and by default’, emerging privacy conserving technologies and contractual controls. Access to protected data is always strictly controlled using legally binding data sharing contracts. UKHSA welcomes data applications from organisations looking to use protected data for public health purposes. To request an application pack or discuss a request for UKHSA data you would like to submit, contact DataAccess@ukhsa.gov.uk.

## Code availability

The code required to run the model introduced in this manuscript and a baseline model using simulated data are available at: 10.5281/zenodo.10023092^57^. The simulated data is representative of the English influenza seasonal wave 2022-2023.
