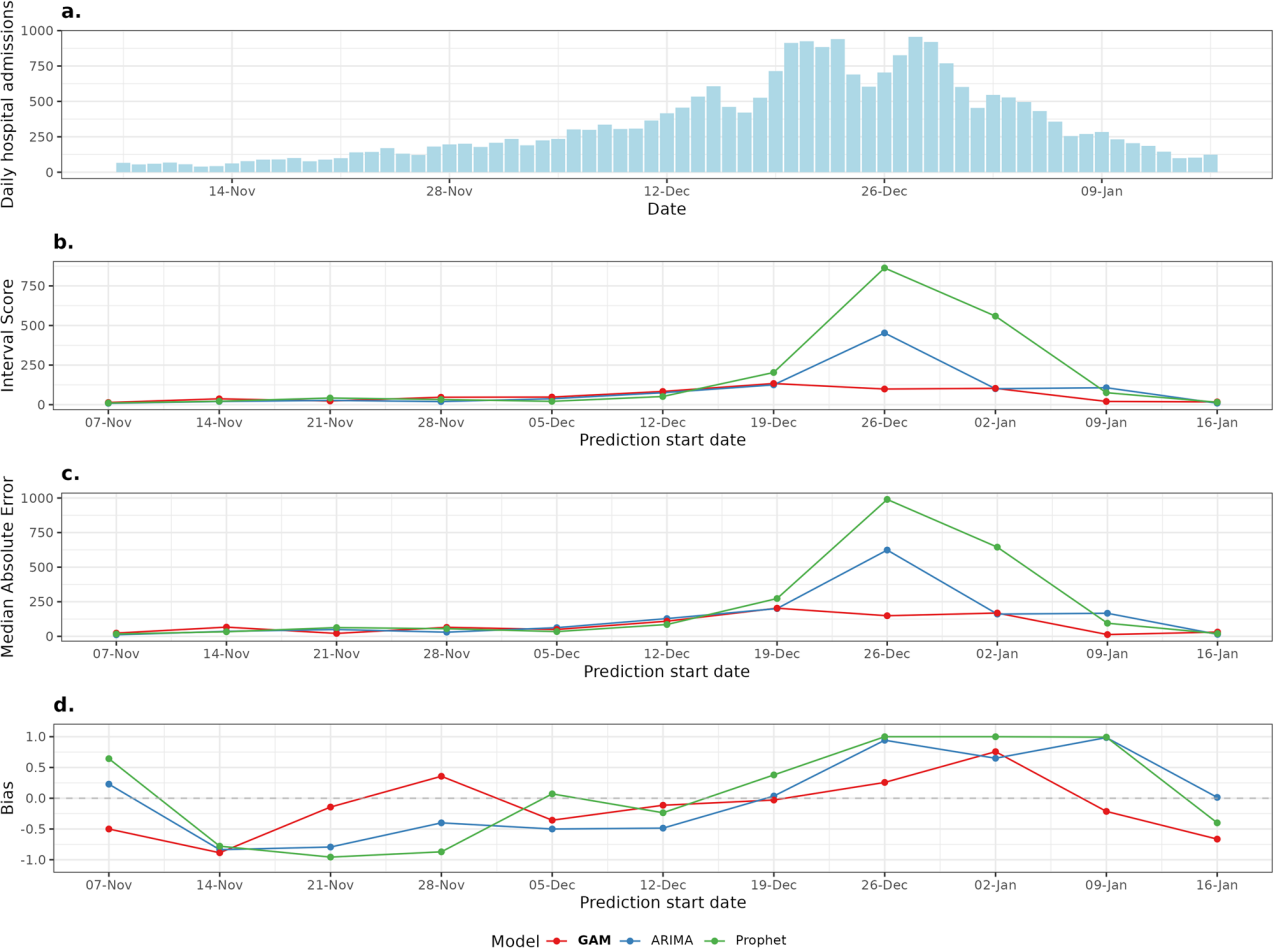# Author Correction: Forecasting influenza hospital admissions within English sub-regions using hierarchical generalised additive models

**DOI:** 10.1038/s43856-024-00435-9

**Published:** 2024-01-30

**Authors:** Jonathon Mellor, Rachel Christie, Christopher E. Overton, Robert S. Paton, Rhianna Leslie, Maria Tang, Sarah Deeny, Thomas Ward

**Affiliations:** 1 UK Health Security Agency, Data Analytics and Surveillance, 10 South Colonnade, London, UK; 2 University of Liverpool, Department of Mathematical Sciences, Liverpool, UK

**Keywords:** Influenza virus, Epidemiology

Correction to: *Communications Medicine* 10.1038/s43856-023-00424-4, published online 20 December 2023.

In this article, Figs. 1 and 4a contained errors in the numbers stated on the x-axis (daily hospital admissions); the figures should have appeared as shown below. The original article has been corrected.

Fig. 1
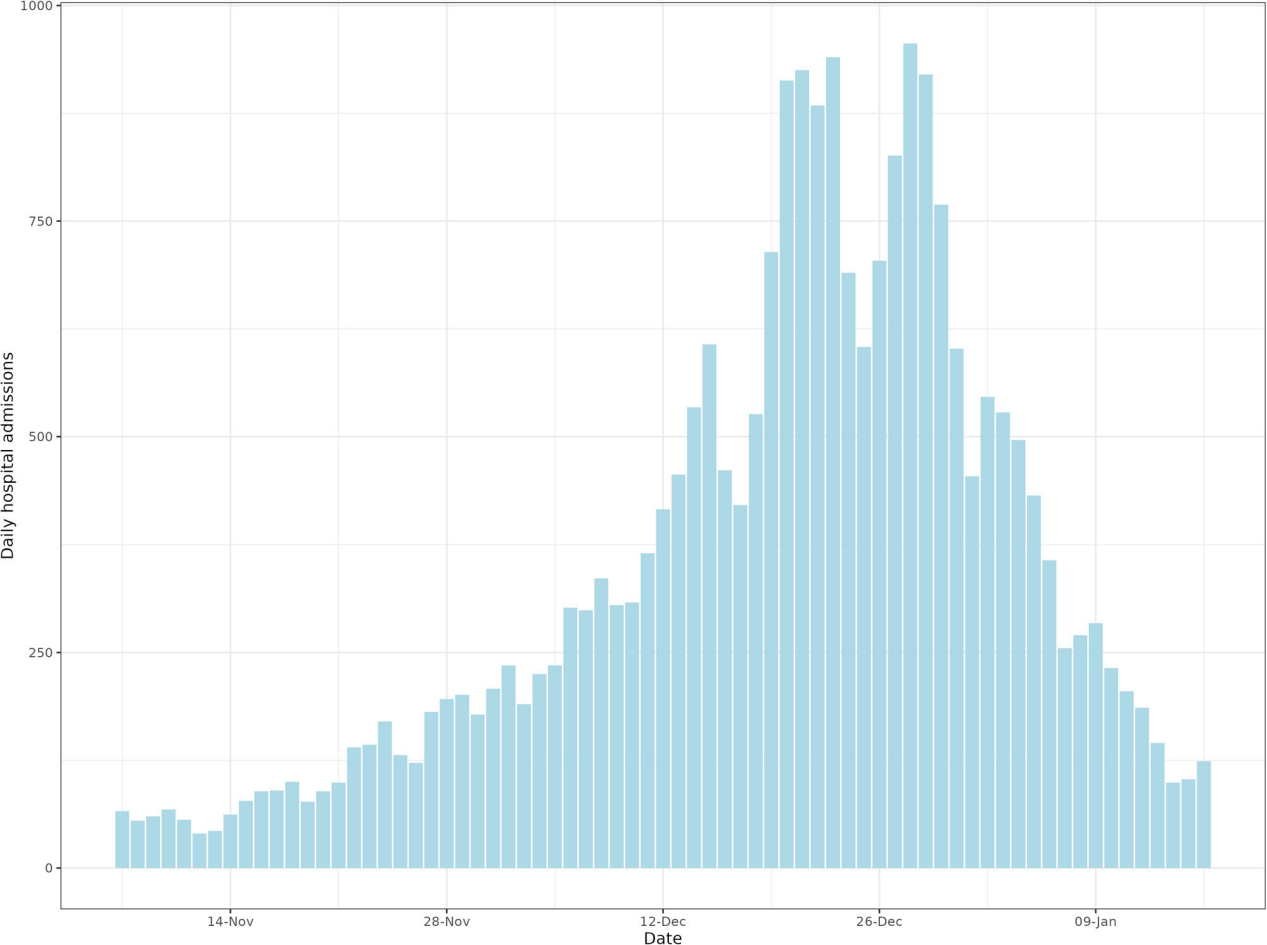


Fig. 4