# Supplementary material for: Forecasting influenza hospital admissions within English sub-regions using hierarchical generalised additive models
Source: Commun Med (Lond). 2023 Dec 20;3:190. doi: 10.1038/s43856-023-00424-4 (PMC10733380; doi:10.1038/s43856-023-00424-4)
Supplement: Supplementary file 1 — Supplementary Information [file 43856_2023_424_MOESM1_ESM.pdf]

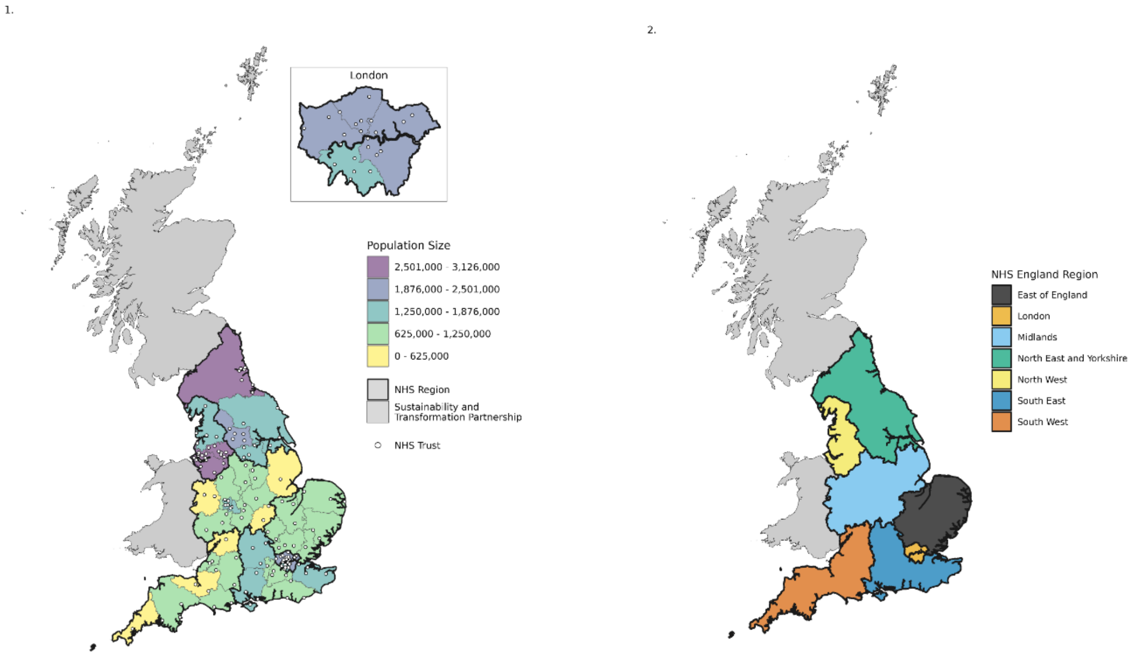

Supplementary Figure 1 A&B. Map of National Health Service (NHS) structure in England (subplot 1). Trust locations were calculated as the modal site postcode of buildings within a Trust. The population size was calculated using a respiratory illness probabilistic mapping from local authority to Trust, aggregated to Sustainability and Transformation Partnerships (STP) level. NHS Commissioning Region boundaries are highlighted across England (subplot 2) to highlight the high-level structures. While not part of this study, Wales and Scotland are illustrated in grey to contextualise the map of England.

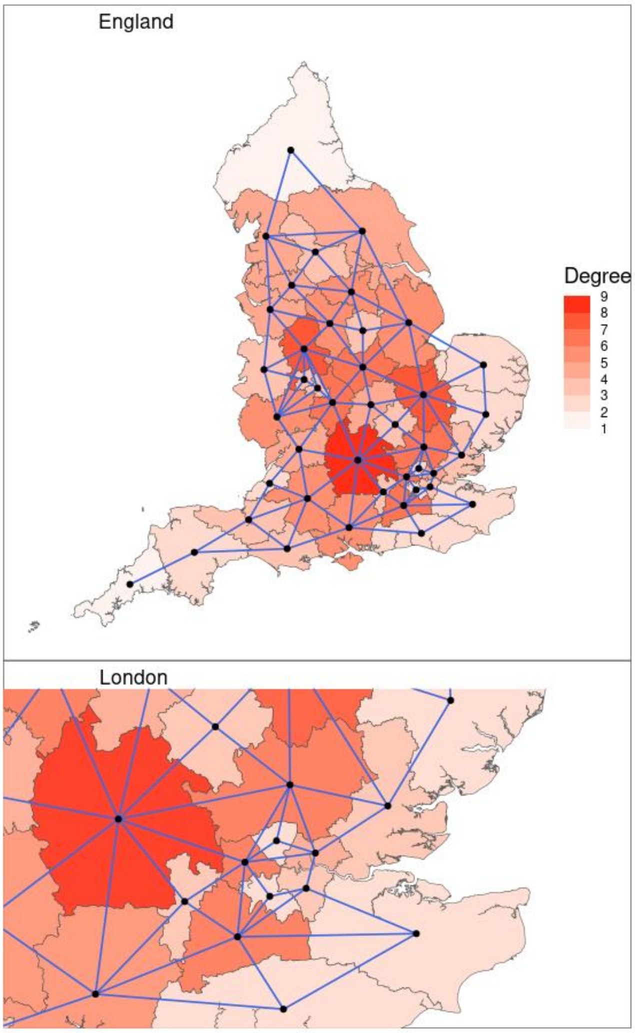

Supplementary Figure 2. A map of the NHS STP boundary polygons and the network constructed by the adjacency of boundaries. The undirected unweighted network is created by connecting adjacent neighbours whose boundaries touch. The network is visualised by connecting the centroids of adjacent STPs. The number of adjacent STPs for a given location is given by the degree, with more red locations having higher degree.

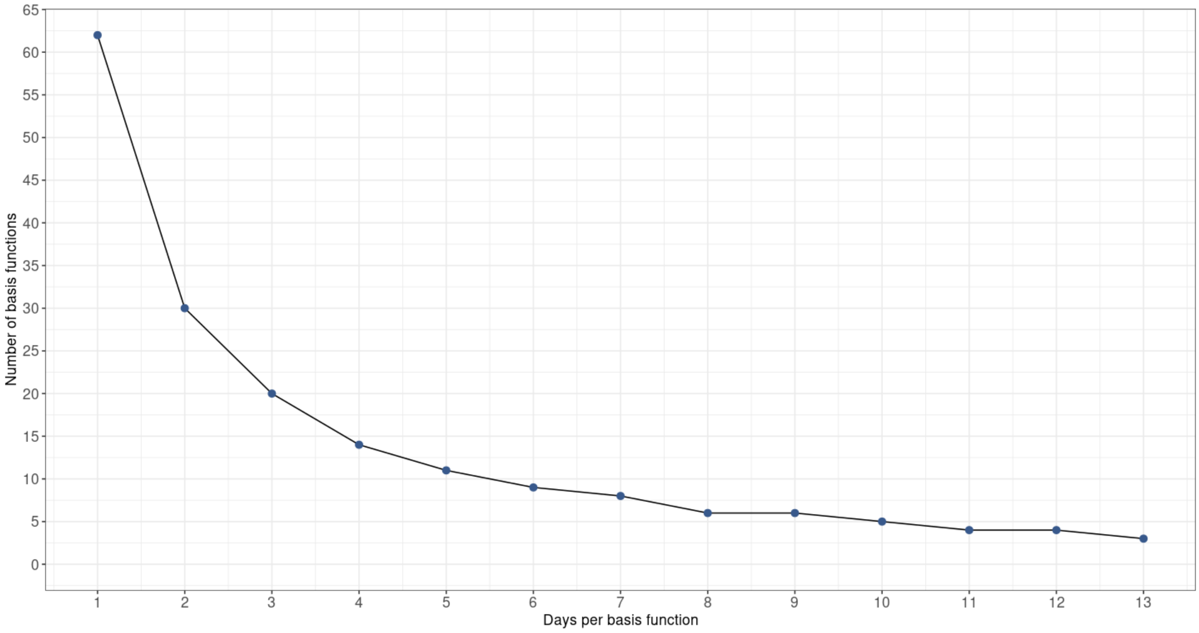

Supplementary Figure 3. Days per basis function and the corresponding number of basis functions chosen for a GAM, with nine weeks of past days fit to.

Evaluation scores for ARIMA and Hierarchical ARIMA models

Model	Horizon	Interval Score	Underprediction	Overprediction	Median Absolute Error	50% Coverage	90% Coverage
National							
ARIMA	Overall	**88.1**	**12.7**	**47.6**	**160**	**0.279**	**0.773**
Hierarchical ARIMA	Overall	167	30.6	115	226	0.149	0.370
ARIMA	7	**71.1**	**8.4**	**39.7**	**146**	**0.000**	**0.727**
Hierarchical ARIMA	7	159	24.9	115	218	**0.000**	0.364
ARIMA	14	**169**	**21.0**	**105**	**294**	**0.182**	**0.545**
Hierarchical ARIMA	14	322	40.6	242	406	**0.182**	0.182
Regional							
ARIMA	Overall	**19.3**	**4.03**	**8.67**	**33.6**	**0.359**	**0.764**
Hierarchical ARIMA	Overall	21.9	5.66	10.7	34.7	0.239	0.567
ARIMA	7	**17.3**	**3.90**	**7.45**	**30.9**	**0.338**	**0.740**
Hierarchical ARIMA	7	20.1	4.81	10.1	33.1	0.130	0.571
ARIMA	14	**35.9**	**6.89**	**18.8**	**58.1**	**0.221**	**0.610**
Hierarchical ARIMA	14	40.9	9.18	22.5	59.5	0.156	0.364

Supplementary Table 1. Interval score, underprediction, overprediction, absolute median error and coverage at 50% and 90% for the baseline ARIMA model and a bottom up hierarchical ARIMA model at national and regional level. The Horizon column denotes how each model scores for the prediction at 7 and 14 days and across all predictions in the two-week forecast, from each week starting 07 Nov 2022 to 16 Jan 2023. The score in bold highlights the best score of the models for each horizon. Bold elements represent the best scoring value for a model within a geography and horizon.

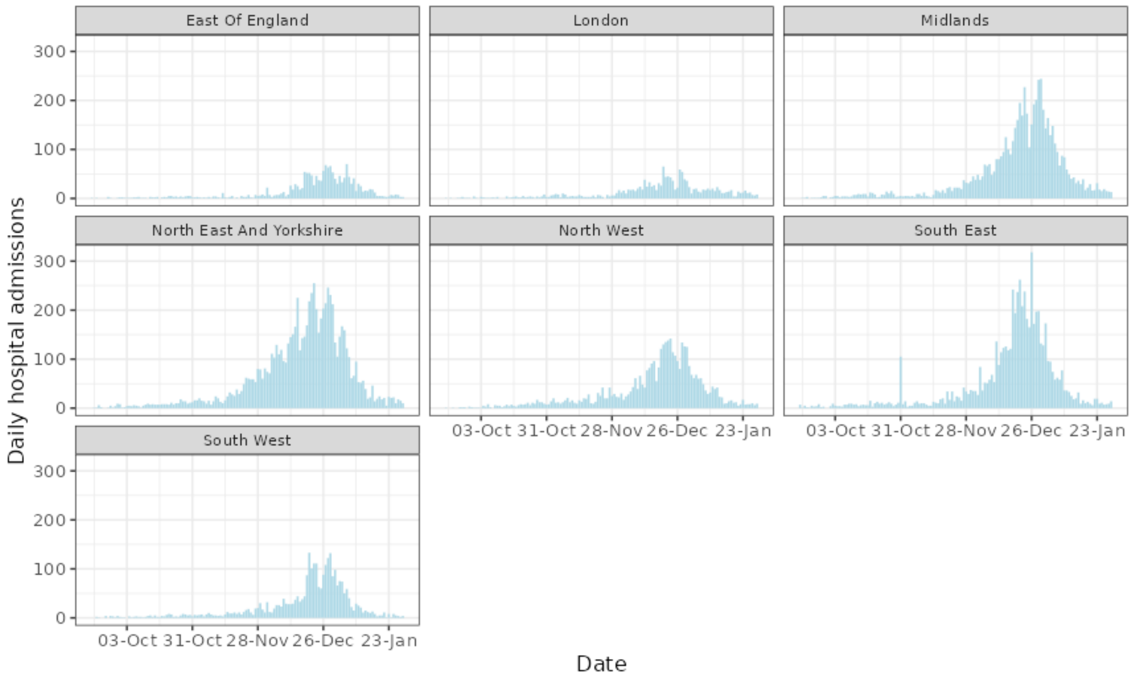

Supplementary Figure 4. Regional influenza 2022/23 season for England between 18 Sept 2022 and 29 Jan 2022, using the NHSE UEC SitRep.

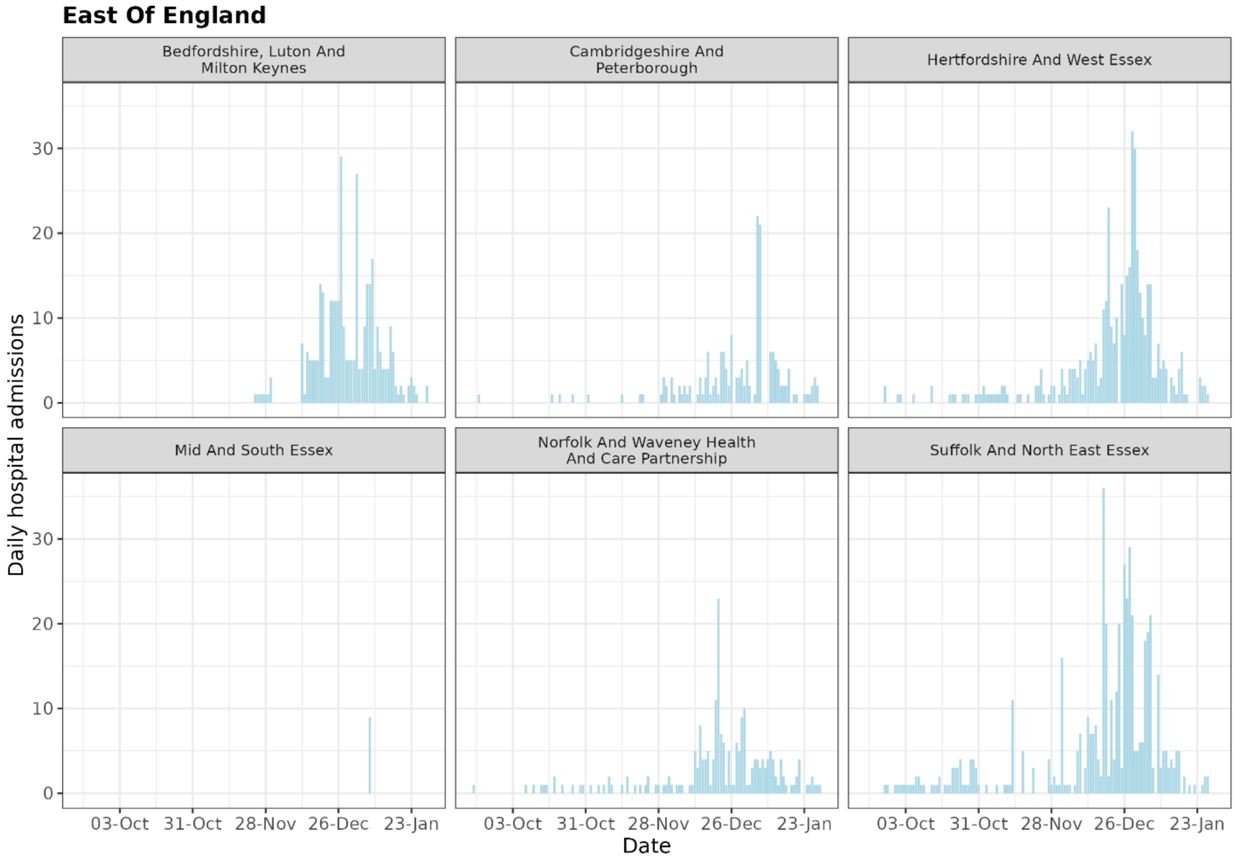

Supplementary Figure 6. East of England influenza 2022/23 season broken down by STP, between 18 Sept 2022 and 29 Jan 2022, using the NHSE UEC SitRep.

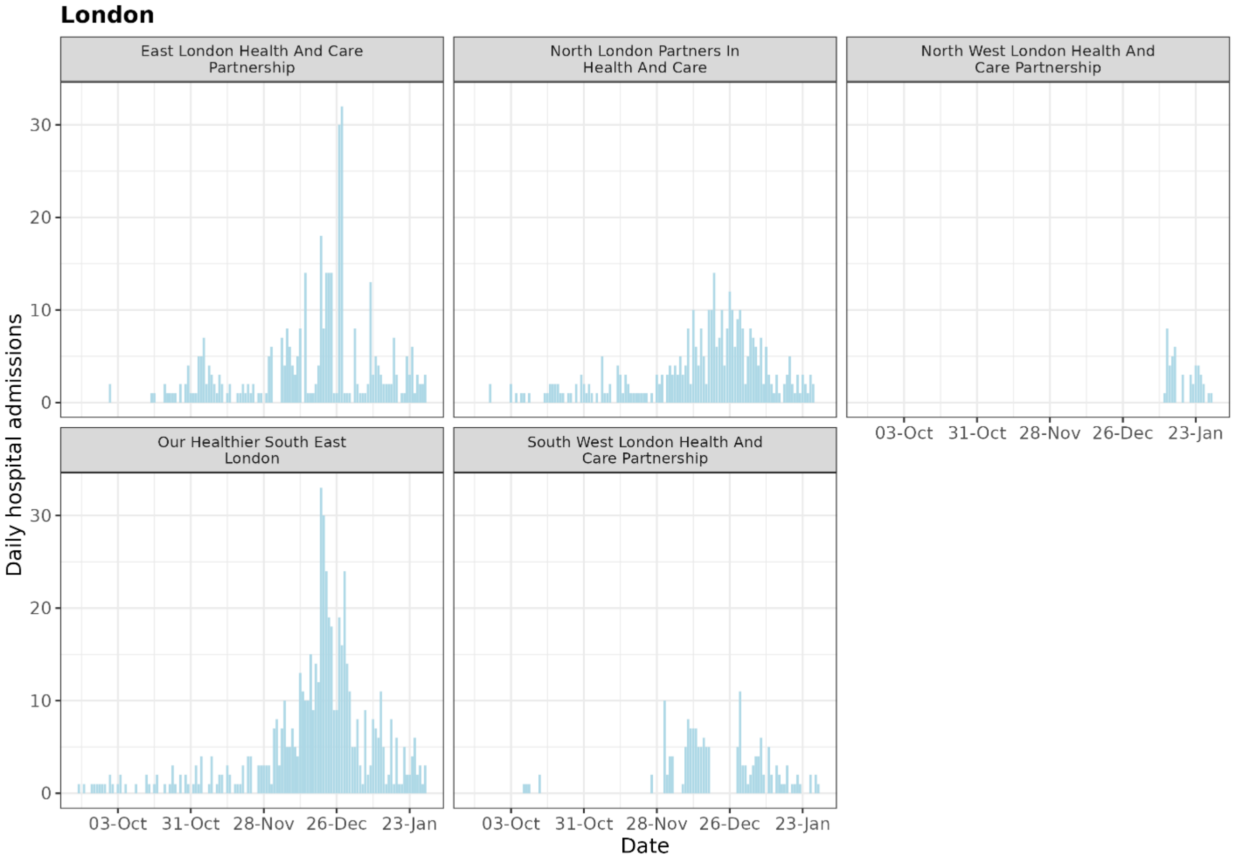

Supplementary Figure 7. London influenza 2022/23 season broken down by STP, between 18 Sept 2022 and 29 Jan 2022, using the NHSE UEC SitRep.

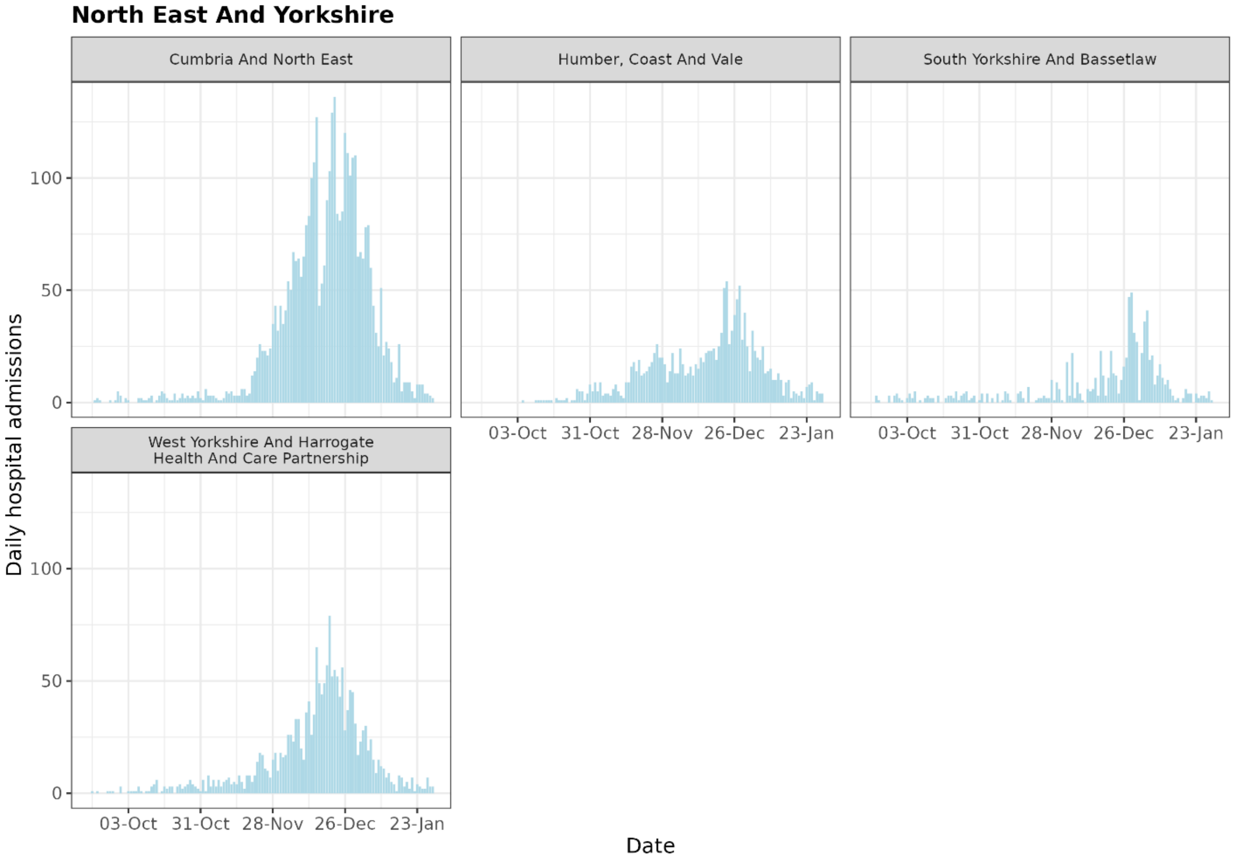

Supplementary Figure 8. North East and Yorkshire influenza 2022/23 season broken down by STP, between 18 Sept 2022 and 29 Jan 2022, using the NHSE UEC SitRep.

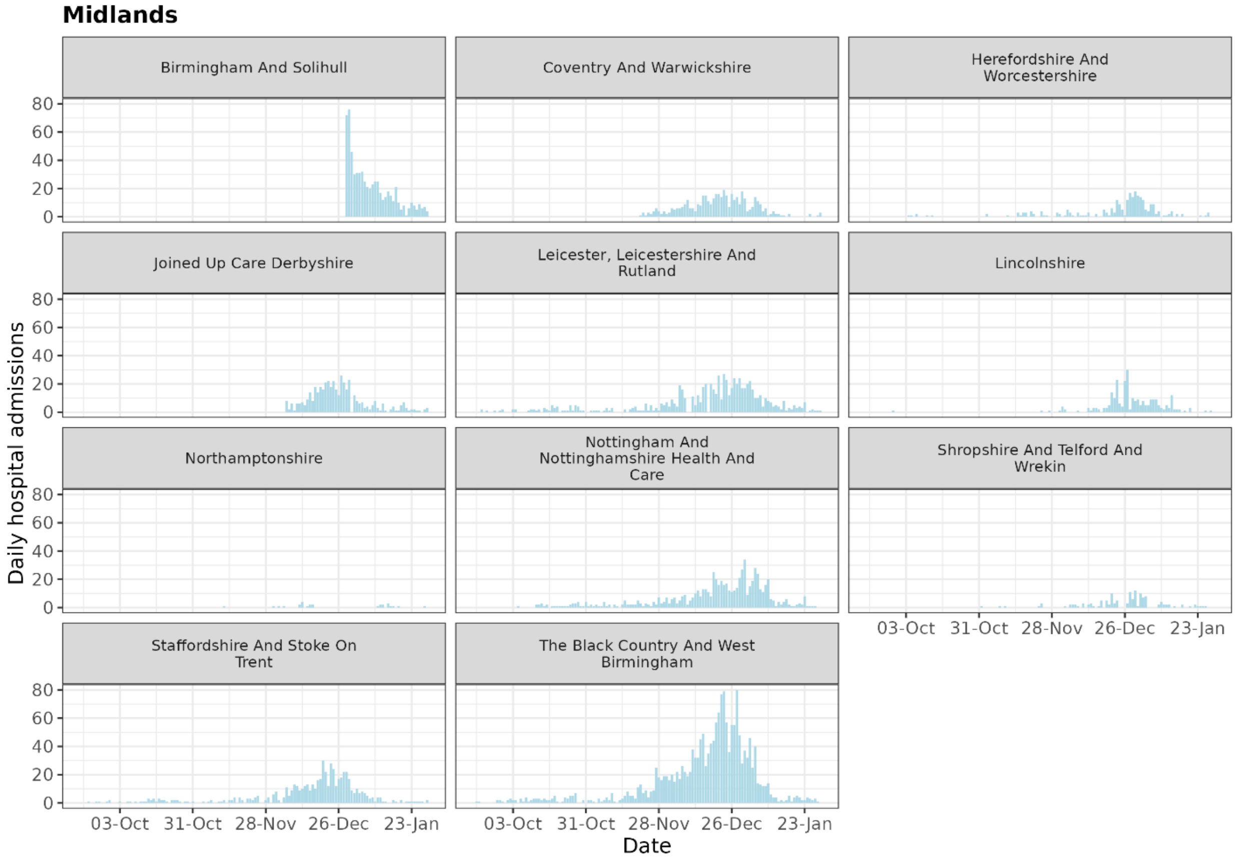

Supplementary Figure 9. Midlands influenza 2022/23 season broken down by STP, between 18 Sept 2022 and 29 Jan 2022, using the NHSE UEC SitRep.

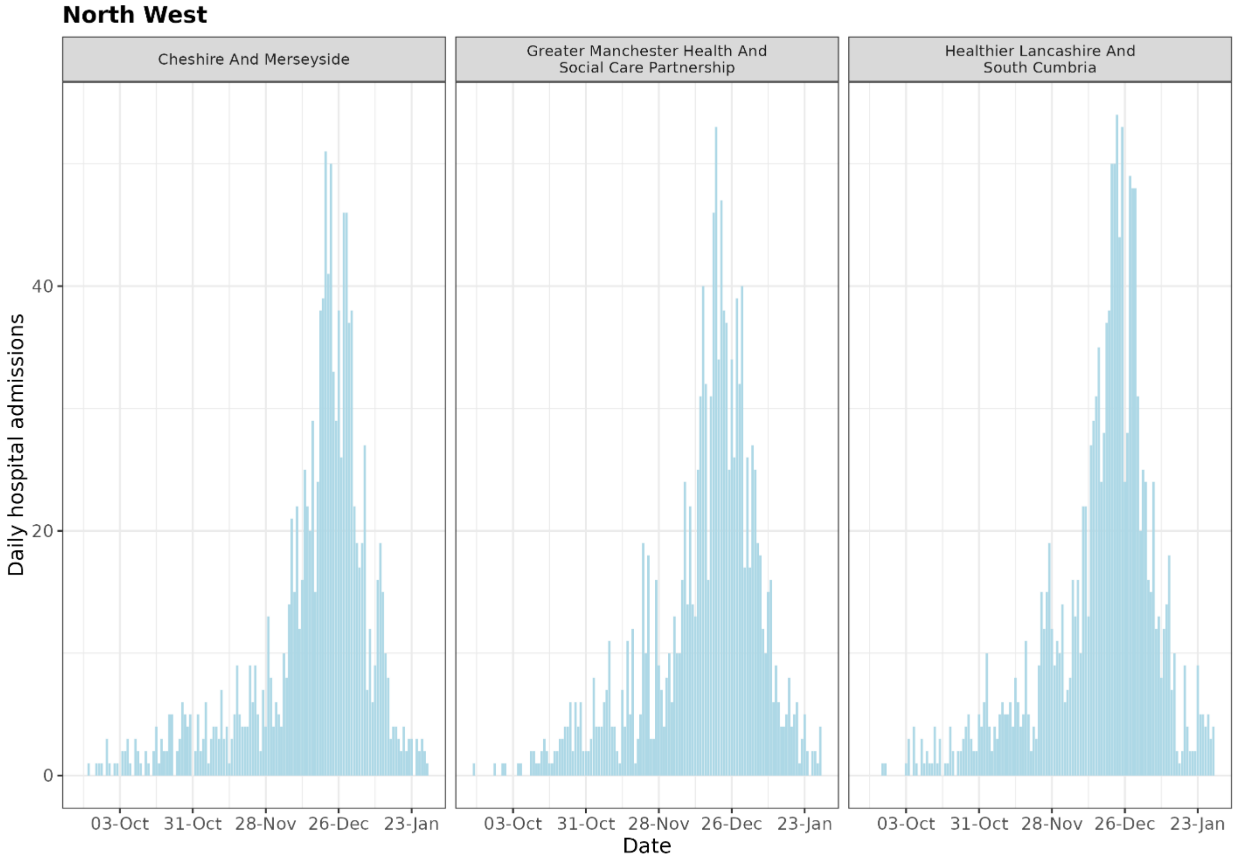

Supplementary Figure 10. North West influenza 2022/23 season broken down by STP, between 18 Sept 2022 and 29 Jan 2022, using the NHSE UEC SitRep.

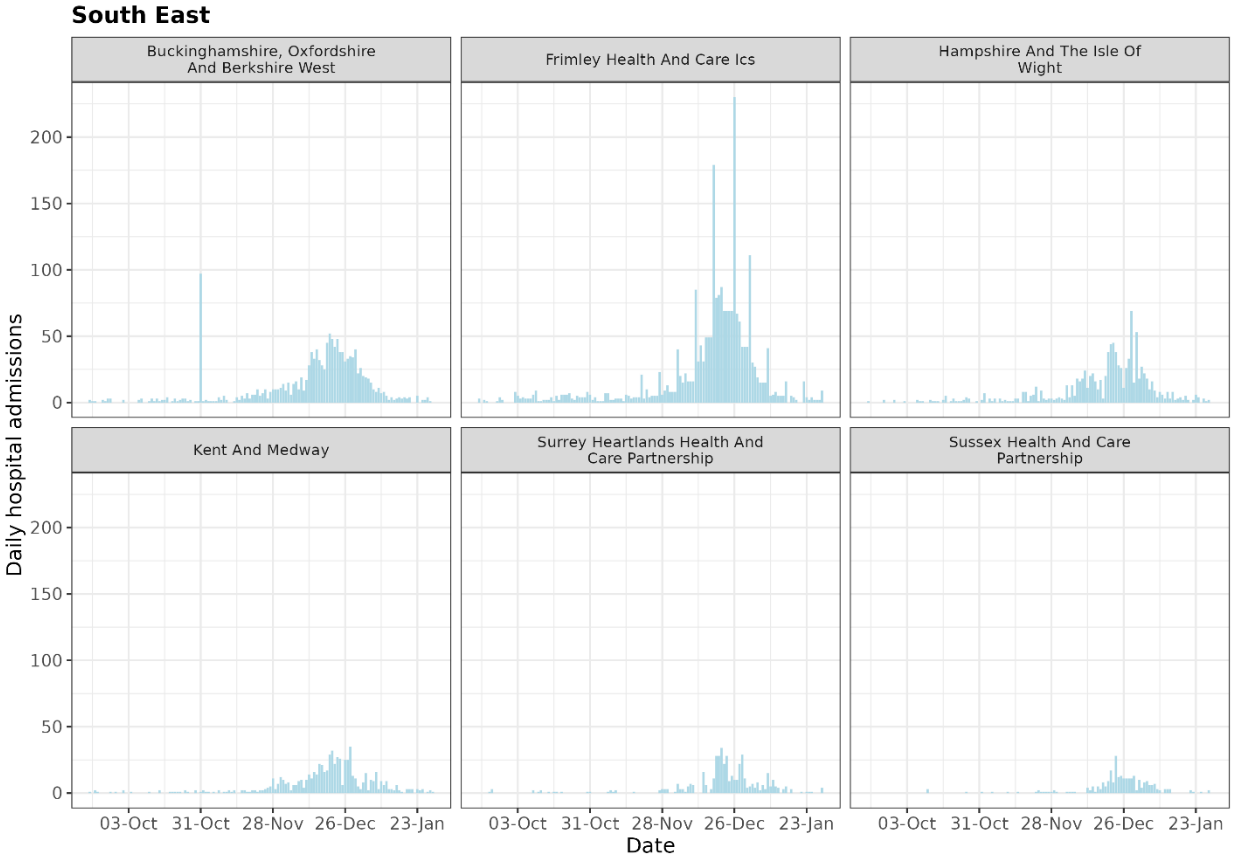

Supplementary Figure 11. South East influenza 2022/23 season broken down by STP, between 18 Sept 2022 and 29 Jan 2022, using the NHSE UEC SitRep.

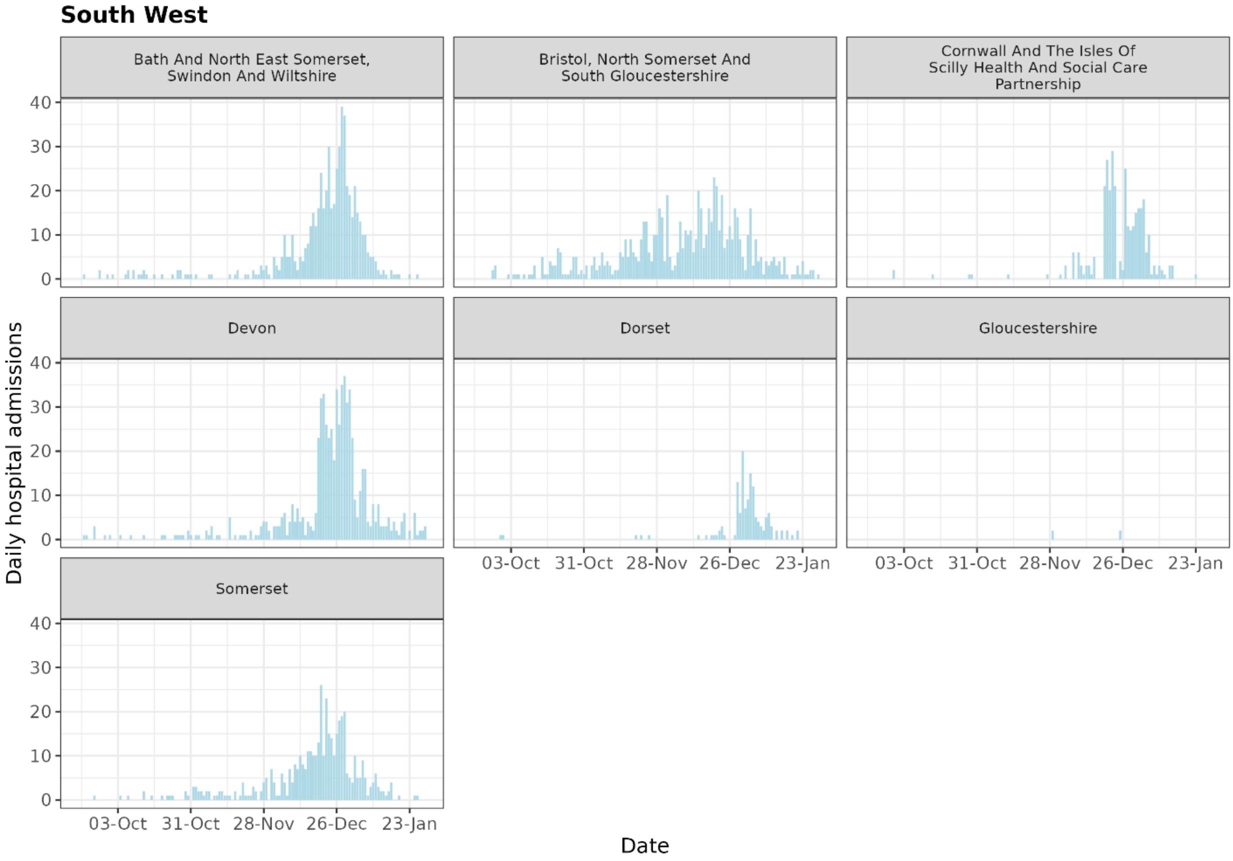

Supplementary Figure 12. South West influenza 2022/23 season broken down by STP, between 18 Sept 2022 and 29 Jan 2022, using the NHSE UEC SitRep.

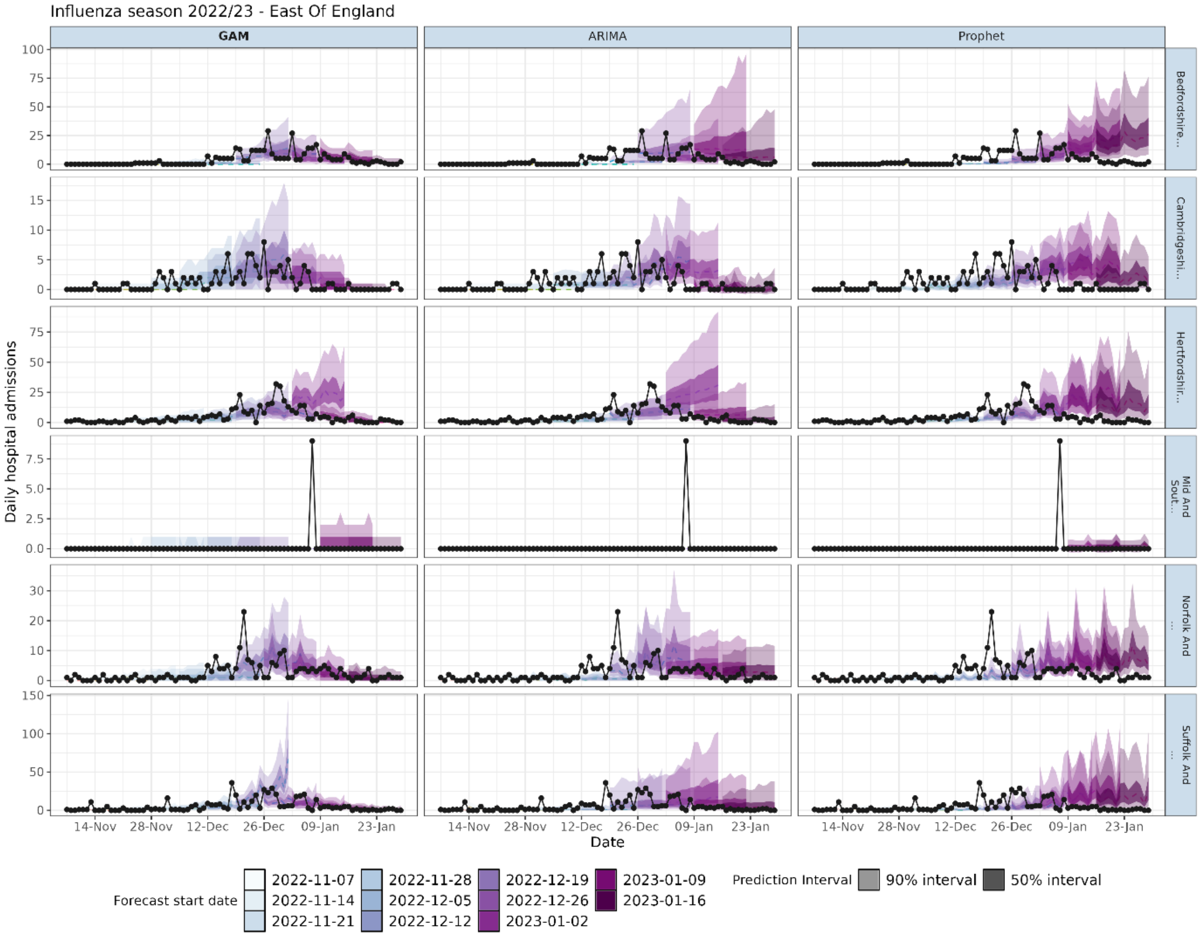

Supplementary Figure 13. Influenza season and model projections for STPs within the East of England. Weekly forecasts of 14 days for the GAM, ARIMA and Prophet models are shown throughout the influenza season. Black dots and lines represent the true admissions for each geographic breakdown.

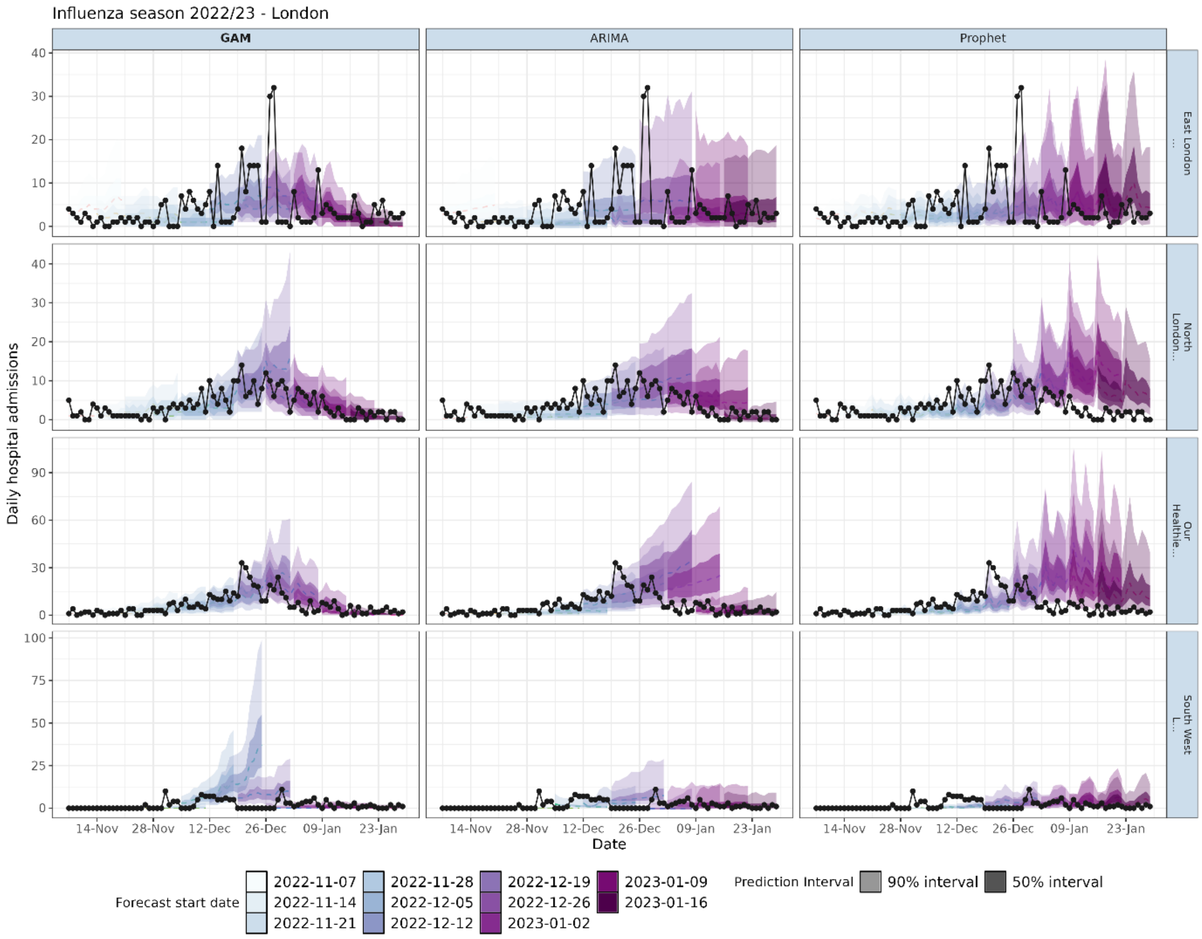

Supplementary Figure 14. Influenza season and model projections for STPs within London. Weekly forecasts of 14 days for the GAM, ARIMA and Prophet models are shown throughout the influenza season. Black dots and lines represent the true admissions for each geographic breakdown.

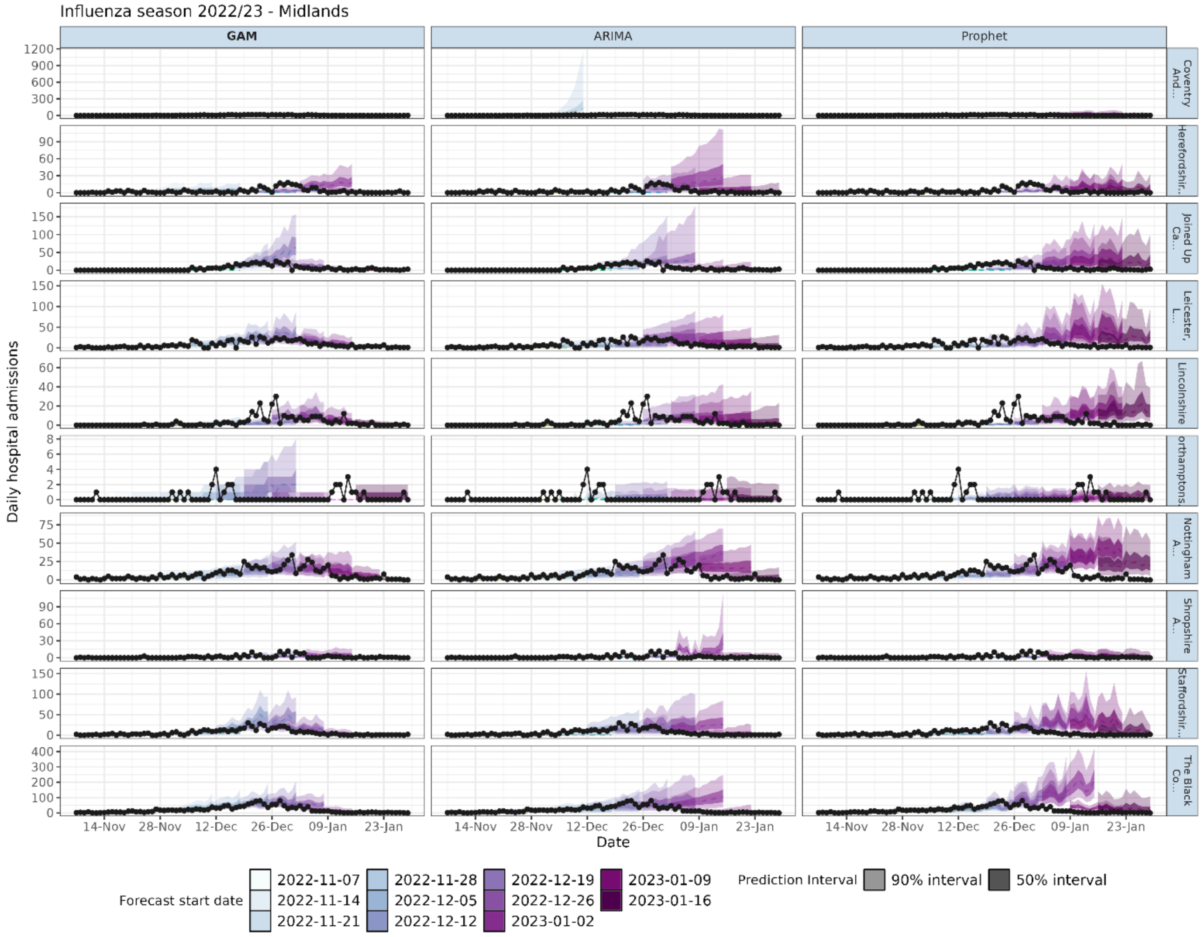

Supplementary Figure 15. Influenza season and model projections for STPs within the Midlands. Weekly forecasts of 14 days for the GAM, ARIMA and Prophet models are shown throughout the influenza season. Black dots and lines represent the true admissions for each geographic breakdown.

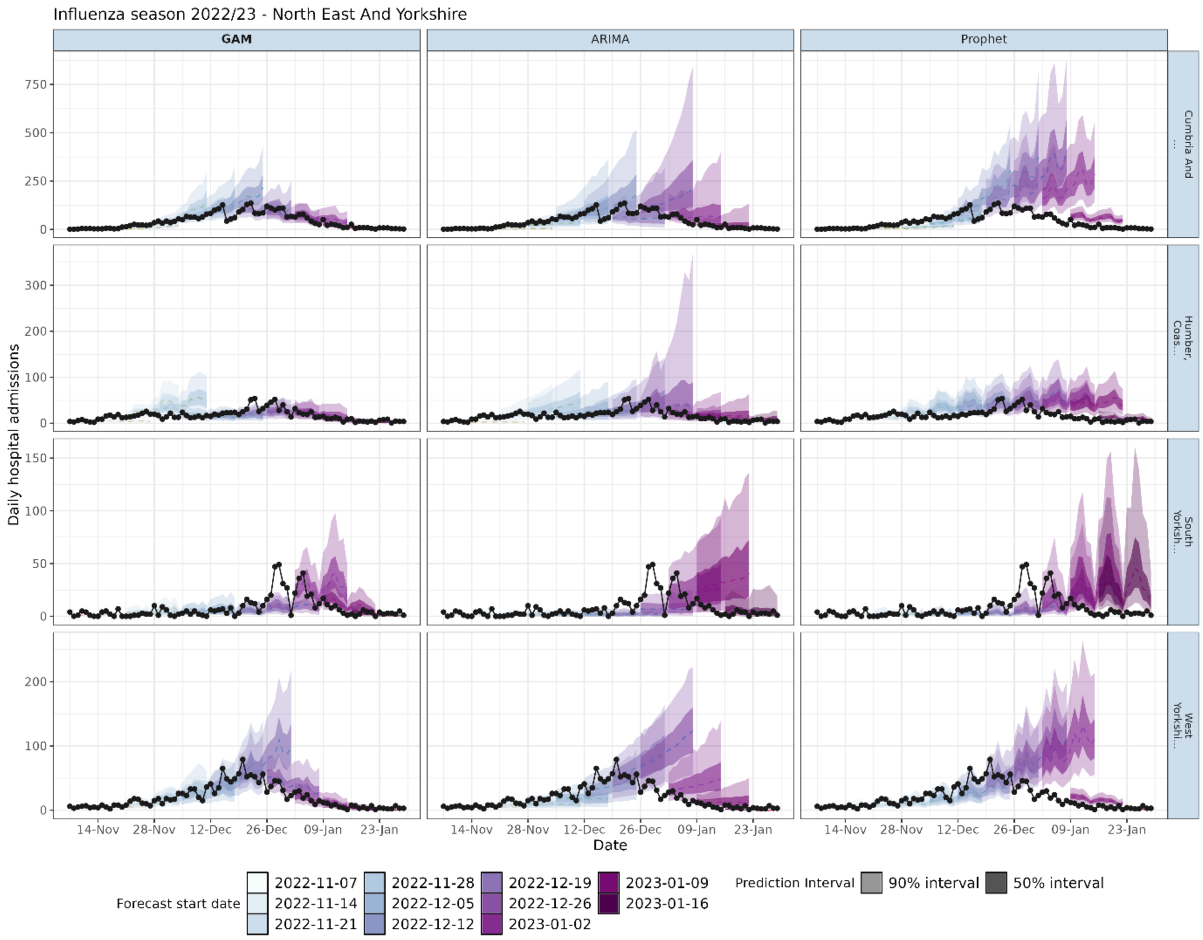

Supplementary Figure 16. Influenza season and model projections for STPs within the North Eat and Yorkshire. Weekly forecasts of 14 days for the GAM, ARIMA and Prophet models are shown throughout the influenza season. Black dots and lines represent the true admissions for each geographic breakdown.

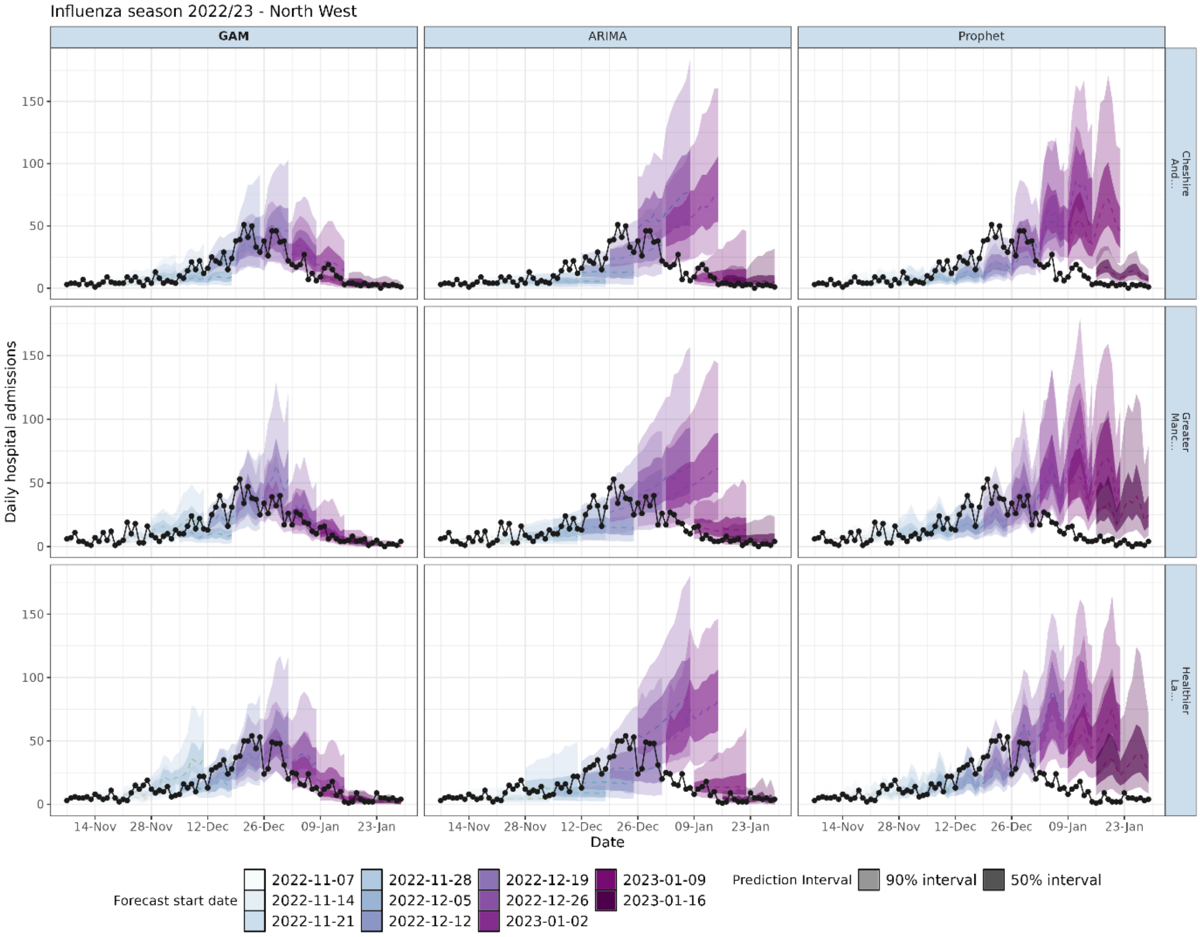

Supplementary Figure 17. Influenza season and model projections for STPs within the North West. Weekly forecasts of 14 days for the GAM, ARIMA and Prophet models are shown throughout the influenza season. Black dots and lines represent the true admissions for each geographic breakdown.

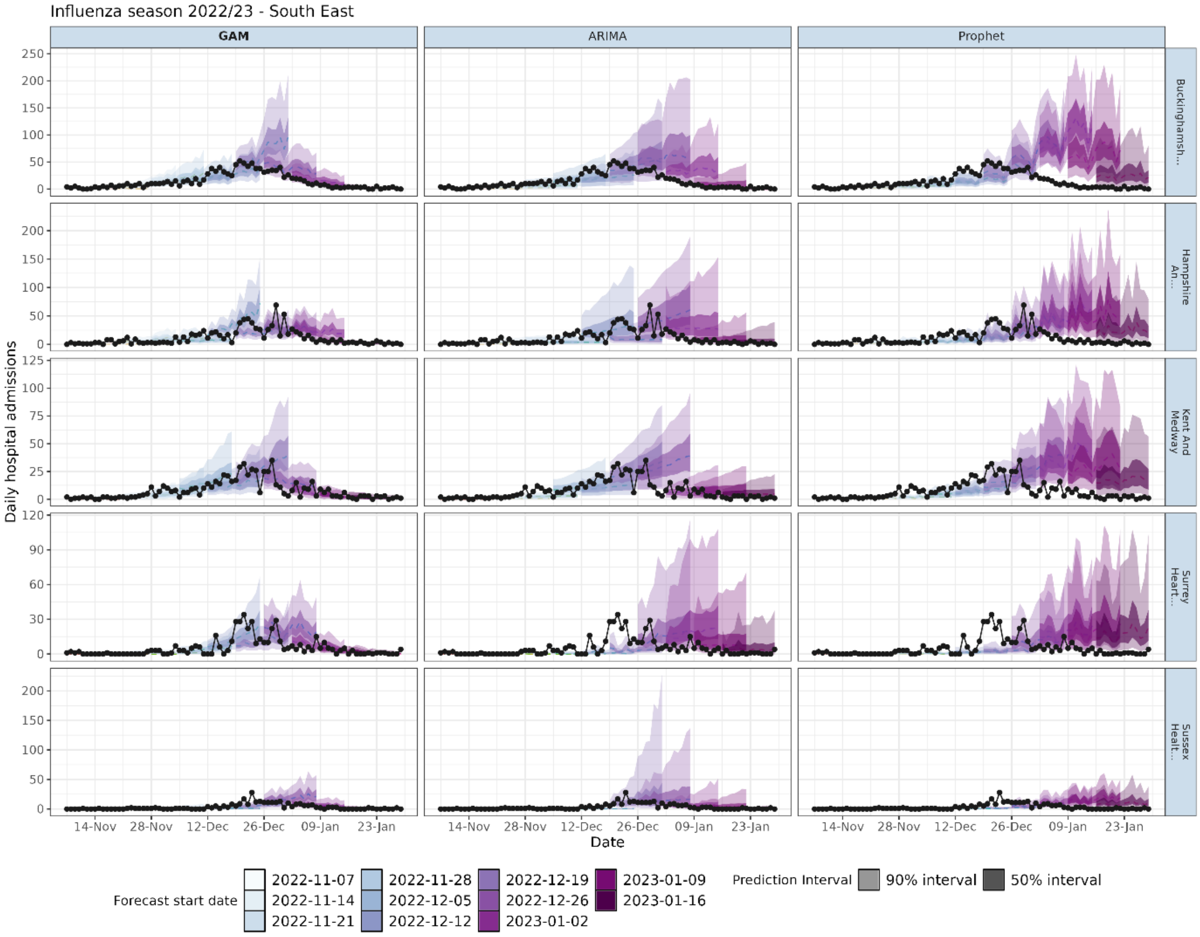

Supplementary Figure 18. Influenza season and model projections for STPs within the South East. Weekly forecasts of 14 days for the GAM, ARIMA and Prophet models are shown throughout the influenza season. Black dots and lines represent the true admissions for each geographic breakdown.

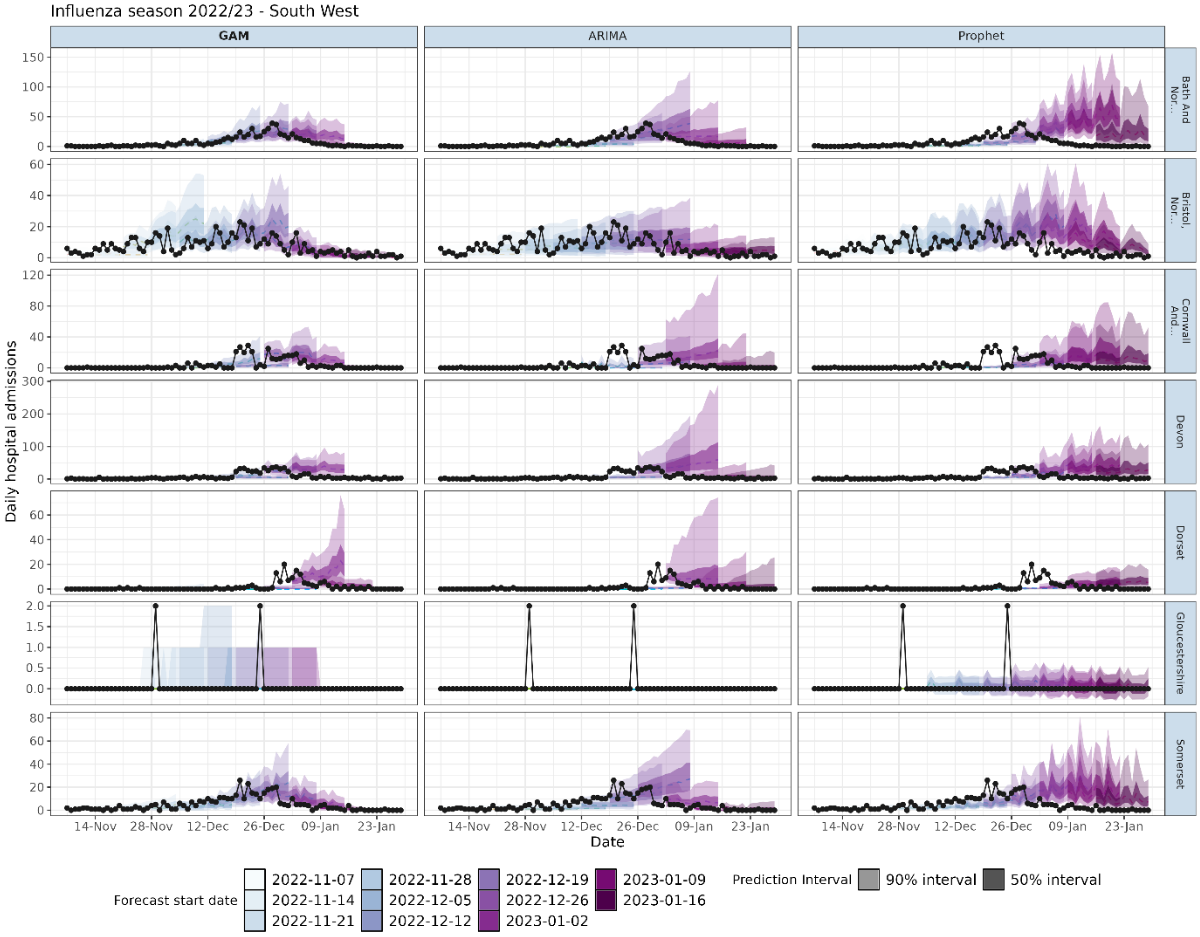

Supplementary Figure 19. Influenza season and model projections for STPs within the South West. Weekly forecasts of 14 days for the GAM, ARIMA and Prophet models are shown throughout the influenza season. Black dots and lines represent the true admissions for each geographic breakdown.

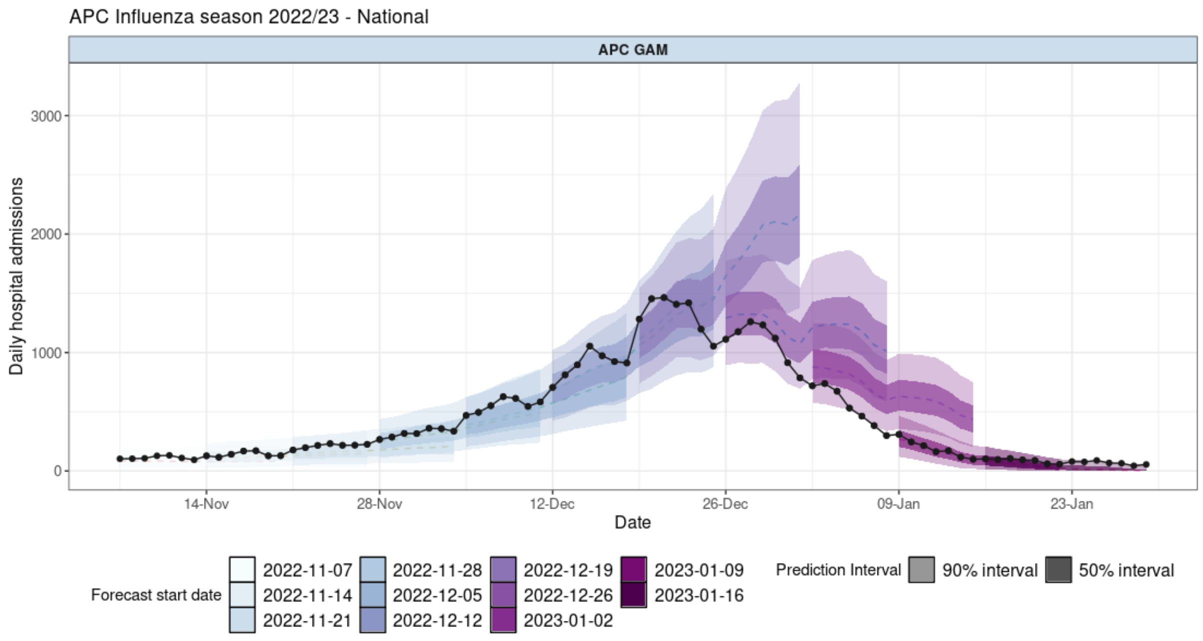

Supplementary Figure 20. National influenza season using Admitted Patient Care (APC) retrospective data and model projections. Weekly forecasts of 14 days for the GAM model are shown throughout the influenza season, using the same tuning parameters as the SitRep GAM. Black dots and lines represent the true admissions nationally. The model over predicts at the peak of the epidemic wave but captures the growth phase and much of the decline phase well.

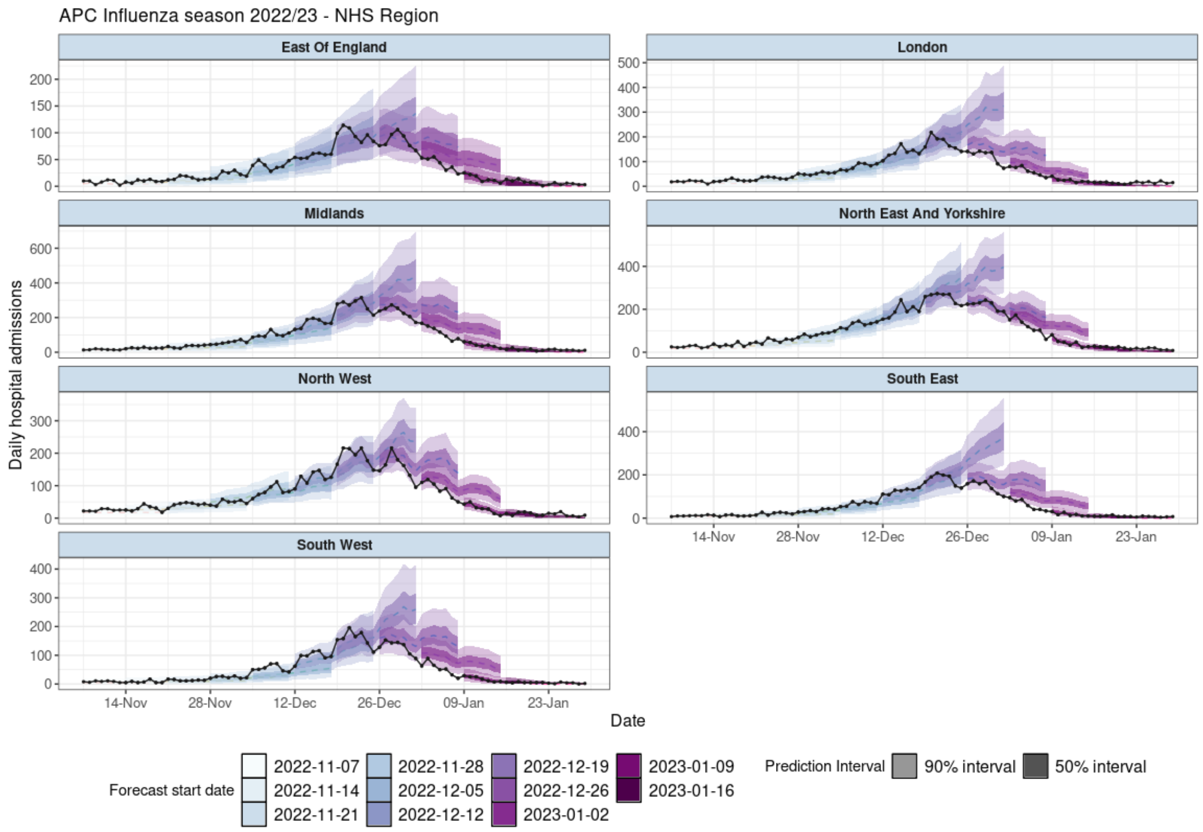

Supplementary Figure 21. Regional influenza season using APC data and model projections. Weekly forecasts of 14 days for the GAM model are shown throughout the influenza season, using the same tuning parameters as the SitRep GAM. Black dots and lines represent the true admissions for each geographic breakdown. As with the other models, the forecasting at the peak of the epidemic is not well predicted, however this could be improved with further tuning to this specific data set.

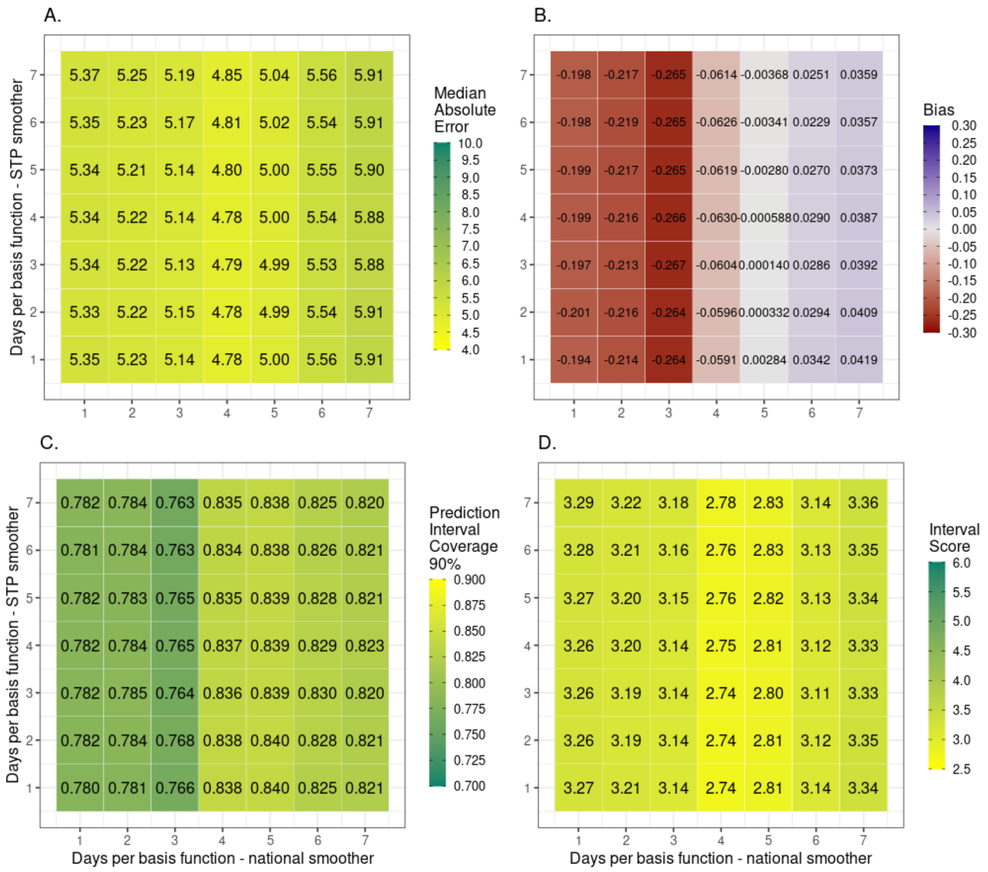

Supplementary Figure 22. The median absolute error (A.), bias (B.), prediction interval coverage (C.) and interval score (D.) metrics for combinations of different numbers of basis functions in the hierarchical GAM. For each metric, a yellow value represents strong relative performance. Models were evaluated on held out forecasting performance for two-week projections, each week from 10 Nov 2022 to 08 Jan 2022.
