# Supplementary material for: Forecasting influenza hospital admissions within English sub-regions using hierarchical generalised additive models
Source: Commun Med (Lond). 2023 Dec 20;3:190. doi: 10.1038/s43856-023-00424-4 (PMC10733380; doi:10.1038/s43856-023-00424-4)
Supplement: Supplementary file 2 — Reporting Summary [file 43856_2023_424_MOESM2_ESM.pdf]

nature portfolio

Corresponding author(s): Jonathon Mellor

Last updated by author(s): Nov 8, 2023

Reporting Summary

Nature Portfolio wishes to improve the reproducibility of the work that we publish. This form provides structure for consistency and transparency in reporting. For further information on Nature Portfolio policies, see our Editorial Policies and the Editorial Policy Checklist.

Statistics

For all statistical analyses, confirm that the following items are present in the figure legend, table legend, main text, or Methods section.

n/a	Confirmed	
☒	☐	The exact sample size (*n*) for each experimental group/condition, given as a discrete number and unit of measurement
☒	☐	A statement on whether measurements were taken from distinct samples or whether the same sample was measured repeatedly
☒	☐	The statistical test(s) used AND whether they are one- or two-sided<br>*Only common tests should be described solely by name; describe more complex techniques in the Methods section.*
☐	☒	A description of all covariates tested
☐	☒	A description of any assumptions or corrections, such as tests of normality and adjustment for multiple comparisons
☐	☒	A full description of the statistical parameters including central tendency (e.g. means) or other basic estimates (e.g. regression coefficient) AND variation (e.g. standard deviation) or associated estimates of uncertainty (e.g. confidence intervals)
☒	☐	For null hypothesis testing, the test statistic (e.g. *F*, *t*, *r*) with confidence intervals, effect sizes, degrees of freedom and *P* value noted<br>*Give P values as exact values whenever suitable.*
☒	☐	For Bayesian analysis, information on the choice of priors and Markov chain Monte Carlo settings
☒	☐	For hierarchical and complex designs, identification of the appropriate level for tests and full reporting of outcomes
☒	☐	Estimates of effect sizes (e.g. Cohen's *d*, Pearson's *r*), indicating how they were calculated

Our web collection on statistics for biologists contains articles on many of the points above.

Software and code

Policy information about availability of computer code

Data collection	No software used to collect this data.
Data analysis	Link to code repository:<br>https://zenodo.org/records/10023092?<br>token=eyJhbGciOiJIUzUxMiJ9.eyJpZCI6ImM5M2ZkOWMyLWJkODAtNDkyMC05ZWMzLTVkOTRmNmNlZDk0ZCIsImRhdGEiOnt9LCJyYW5kb20iOiIwMTBlM2ViZDBhZWQyY2VkNjVkODZlNTljZGRkNmNjZiJ9.P5ujf1oAr0neaj8Z4ZF2lZ3LV_ekzWktO7Dqx5drF-I9ClZncVsvYnfKHmwDjcivK6lWszQS9ucnPgPNKYDIAQ

For manuscripts utilizing custom algorithms or software that are central to the research but not yet described in published literature, software must be made available to editors and reviewers. We strongly encourage code deposition in a community repository (e.g. GitHub). See the Nature Portfolio guidelines for submitting code & software for further information.

Data

Policy information about availability of data

All manuscripts must include a data availability statement. This statement should provide the following information, where applicable:

- Accession codes, unique identifiers, or web links for publicly available datasets
- A description of any restrictions on data availability
- For clinical datasets or third party data, please ensure that the statement adheres to our policy

The data used within this study relies on a range of public and third party data sets. Restrictions apply to the NHSE UEC SitRep data from NHS England which were used under license for this study, and is therefore not publicly available. Other data sets, such as the ONS STP boundary files are available here: https://geoportal.statistics.gov.uk/datasets/ons::sustainability-and-transformation-partnerships-april-2021-en-bfc-3/about. The population estimates were obtained from: https://www.ons.gov.uk/peoplepopulationandcommunity/populationandmigration/populationestimates/datasets/populationestimatesforukenglandandwalesscotlandandnorthernireland

Human research participants

Policy information about studies involving human research participants and Sex and Gender in Research.

Reporting on sex and gender	Sex and gender were not recorded or linkable with this data set.
Population characteristics	Patient characteristics were not recorded or linkable in this study.
Recruitment	Patients were not recruited to this study, administrative counts of patients were recorded as part of operational management within the healthcare systems.
Ethics oversight	UKHSA

Note that full information on the approval of the study protocol must also be provided in the manuscript.

Field-specific reporting

Please select the one below that is the best fit for your research. If you are not sure, read the appropriate sections before making your selection.

☒ Life sciences ☐ Behavioural & social sciences ☐ Ecological, evolutionary & environmental sciences

For a reference copy of the document with all sections, see nature.com/documents/nr-reporting-summary-flat.pdf

Life sciences study design

All studies must disclose on these points even when the disclosure is negative.

Sample size	The sample size was 137 acute hospital trusts within NHS England.
Data exclusions	Data exclusions are outlined in the supplementary section of the paper in further detail. Individual trusts were excluded if their reporting across metrics did not match, or they provided null returns.
Replication	Forecast results were verified against future data points, and validated against a secondary surveillance source for influenza hospitalizations - SARI-Watch. The method is replicated with SUS APC in the paper.
Randomization	Not applicable.
Blinding	Not applicable.

Reporting for specific materials, systems and methods

We require information from authors about some types of materials, experimental systems and methods used in many studies. Here, indicate whether each material, system or method listed is relevant to your study. If you are not sure if a list item applies to your research, read the appropriate section before selecting a response.

Materials & experimental systems

n/a	Involved in the study
☒	☐ Antibodies
☒	☐ Eukaryotic cell lines
☒	☐ Palaeontology and archaeology
☒	☐ Animals and other organisms
☒	☐ Clinical data
☒	☐ Dual use research of concern

Methods

n/a	Involved in the study
☒	☐ ChIP-seq
☒	☐ Flow cytometry
☒	☐ MRI-based neuroimaging
